# A genetic optimization strategy with generality in asymmetric organocatalysis as a primary target†

**DOI:** 10.1039/d3sc06208b

**Published:** 2024-01-31

**Authors:** Simone Gallarati, Puck van Gerwen, Ruben Laplaza, Lucien Brey, Alexander Makaveev, Clemence Corminboeuf

**Affiliations:** a Laboratory for Computational Molecular Design, Institute of Chemical Sciences and Engineering, Ecole Polytechnique Fédérale de Lausanne (EPFL) 1015 Lausanne Switzerland clemence.corminboeuf@epfl.ch; b National Center for Competence in Research – Catalysis (NCCR-Catalysis), Ecole Polytechnique Fédérale de Lausanne (EPFL) 1015 Lausanne Switzerland; c National Center for Computational Design and Discovery of Novel Materials (MARVEL), Ecole Polytechnique Fédérale de Lausanne (EPFL) 1015 Lausanne Switzerland

## Abstract

A catalyst possessing a broad substrate scope, in terms of both turnover and enantioselectivity, is sometimes called “general”. Despite their great utility in asymmetric synthesis, truly general catalysts are difficult or expensive to discover *via* traditional high-throughput screening and are, therefore, rare. Existing computational tools accelerate the evaluation of reaction conditions from a pre-defined set of experiments to identify the most general ones, but cannot generate entirely new catalysts with enhanced substrate breadth. For these reasons, we report an inverse design strategy based on the open-source genetic algorithm NaviCatGA and on the OSCAR database of organocatalysts to simultaneously probe the catalyst and substrate scope and optimize generality as a primary target. We apply this strategy to the Pictet–Spengler condensation, for which we curate a database of 820 reactions, used to train statistical models of selectivity and activity. Starting from OSCAR, we define a combinatorial space of millions of catalyst possibilities, and perform evolutionary experiments on a diverse substrate scope that is representative of the whole chemical space of tetrahydro-β-carboline products. While privileged catalysts emerge, we show how genetic optimization can address the broader question of generality in asymmetric synthesis, extracting structure–performance relationships from the challenging areas of chemical space.

## Abstract 2

A genetic optimization strategy to discover asymmetric organocatalysts with high activity and enantioselectivity across a broad substrate scope.

## Graphical abstract

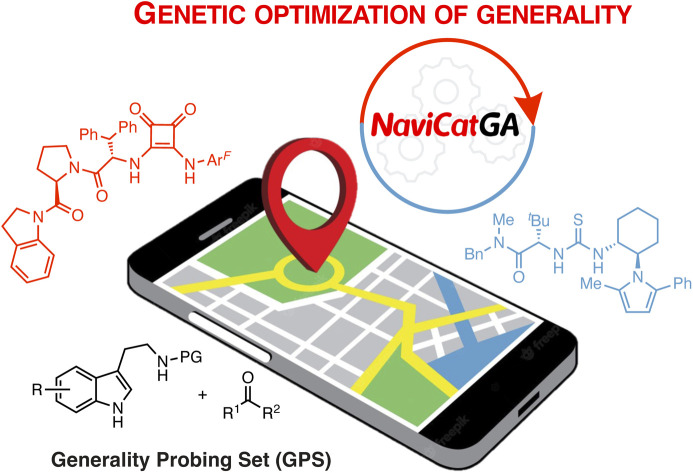

## Introduction

**Fig. 1:**
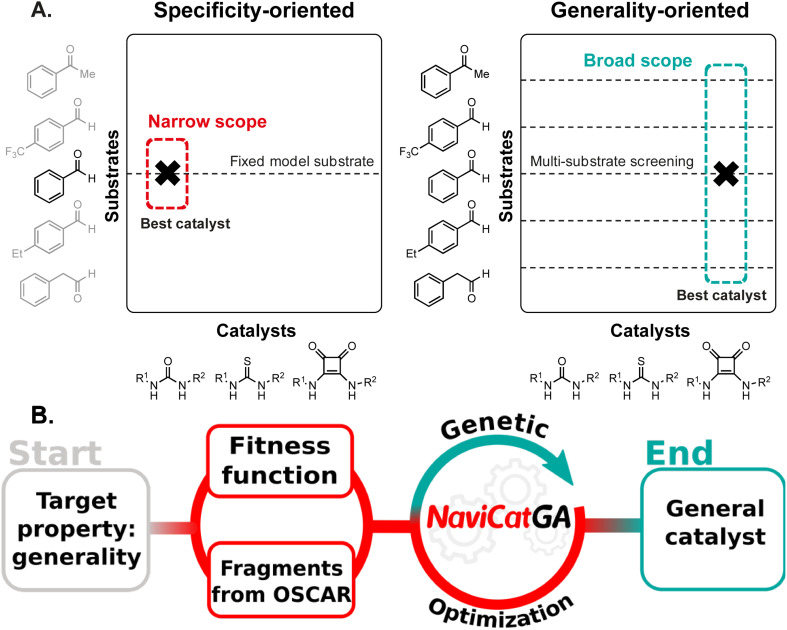
(A) Reaction optimization tactics for the development of catalytic methods: traditional specificity-oriented *vs.* data-driven multi-substrate screening. (B) Schematic inverse design pipeline powered by NaviCatGA.

Developing catalytic methods that are tolerant to many functional groups exerting different steric and electronic influences on the reaction center without significant reduction in yield or product selectivity is a long-standing goal of organic chemistry. Despite being a highly desired feature, such “generality” *i.e.*, breadth of substrate scope,^1^ is rare and only a few transformations become routinely incorporated into the synthetic chemist's toolbox.^2,3^ This is due to reaction development usually beginning with the examination of a simple, readily available model substrate (Fig. 1A), with subsequent re-optimization on more complex systems guided by empirical trial-and-error.^4^ Finding species with enhanced substrate breadth requires evaluating wider regions of chemical space derived from a large matrix of diverse catalysts crossed with a panel of substrates that effectively represent the whole target molecule class. Today, “one-pot-multisubstrate” screening^5–7^ is tractable with high-throughput experimentation techniques,^8–12^ but has found limited applicability due to issues associated with chemical compatibility and product analysis. The catalyst space investigated remains limited, at best, to tens of candidates and, perhaps worse, the most general ones might be unwittingly excluded from the original screening set, biasing the results.^13^

In the last decade, data-driven computational methods, in tandem with supervised and unsupervised machine learning algorithms, have been applied to address numerous challenges in organic chemistry,^14–17^ such as prediction of reaction outcomes,^18–20^ multistep synthetic planning,^21–23^ and catalyst discovery.^24–28^ In particular, Bayesian optimization^29,30^ has been combined with robotic experimentation to find general conditions for heteroaryl Suzuki–Miyaura coupling.^31^ Denmark and co-workers have developed a “catalyst selection by committee” to identify general disulfonimides for the atroposelective iodination of a variety of 2-amino-6-arylpyridines,^26^ and used active learning to provide substrate-adaptive conditions for C–N couplings.^32^ Recently, Reid *et al.* have proposed a workflow for assigning and predicting generality through clustering of reaction sets, but manually curated literature databases and a user-defined success value were required.^33^ Overall, existing data-driven tools are still aimed at accelerating the evaluation of a pre-defined set of catalysts,^34^ rather than suggesting entirely new species exhibiting high performance across the whole substrate scope.

Generative models^35^ are an attractive alternative to direct screening by enabling the inverse design of functional molecules and materials.^36,37^ In this paradigm, the desired functionality (*i.e.*, the target) is first defined, and chemical structures tailored to that property are suggested (Fig. 1B). Although applications of generative models, such as genetic algorithms,^38^ to homogeneous catalysis are increasingly being reported,^39–44^ only specificity-oriented catalyst design has been addressed. Optimizing generality as primary target requires adapting existing tools and pipelines to tackle this multi-dimensional problem.

Here, we show how evolutionary experiments performed with the genetic algorithm NaviCatGA,^45^ leveraging the recently reported OSCAR database of organocatalysts' building blocks,^46^ are designed to simultaneously probe the catalyst and substrate space and find candidates predicted to exhibit both high turnover and enantioselectivity. We discuss the nature of fitness function used to estimate how close candidate species are to achieving optimal performance, the surrogate models that accelerate fitness evaluation, the database of molecular fragments to generate millions of prospective catalysts on-the-fly, and the strategy followed to choose an unbiased and diverse substrate scope. We select the Pictet–Spengler condensation as a synthetically relevant case study to illustrate how multi-objective genetic optimization across an expansive substrate space affords organocatalysts with good median activity and selectivity, while simultaneously providing information rich data on the areas of chemical space where even the best candidates are underperforming. Analysis of the challenging substrates gives insights into the set of non-covalent interactions that are necessary for generality, and into the structural features of the tetrahydro-β-carboline intermediate that disrupt them. Our pipeline allows us to automatically generate candidates with the broadest scope possible, and also to understand why truly “privileged” organocatalysts across highly diverse substrates are difficult to discover.

## Methods: the NaviCatGA pipeline and components

NaviCatGA is a versatile genetic algorithm capable of optimizing homogeneous catalysts by exploiting any suitable fitness function that describes their catalytic performance.^45^ It manipulates catalyst structures generated on-the-fly from a user-defined library of building blocks (*e.g.*, organocatalysts' scaffolds and substituents from OSCAR^46^) using any molecular representation, including SMILES strings and *XYZ* coordinates. By performing an iterative sequence of genetic operations (fitness evaluation, crossover, and mutation), NaviCatGA quickly finds the combination of building blocks that maximizes the fitness function (Fig. 1B).^38^ The role of the fitness function is evaluating how close a potential catalyst is to achieving optimal performance. In the context of asymmetric catalysis, a good catalyst is both enantioselective (*i.e.*, high enantiomeric excess, often converted to ΔΔ*G*^‡^, values) and active (*i.e.*, high percentage yield, or turnover frequency, TOF). Measures of selectivity and activity can be obtained either from experiments or computations. Experimental ΔΔ*G*^‡^ values are notoriously difficult to reproduce accurately with computations,^47^ while experimental yields, especially in the context of asymmetric organocatalysis, are often not reported (or only high-yielding reactions are reported, see Fig. S1 and S2† for further details).^48^ During the evolutionary experiment, the structure of new, untested catalyst candidates is generated, and their fitness must be evaluated: this constitutes the bottleneck of genetic optimization.

For these reasons, herein we adopt a hybrid strategy to evaluate catalyst performance: we (1) exploit experimental ΔΔ*G*^‡^ values curated from the literature to train a statistical model and predict the enantioselectivity of untested catalyst–substrate combinations, and (2) perform DFT computations to construct molecular volcano plots^49–51^ and estimate a catalyst's TOF *via* a descriptor variable, training a second surrogate model of activity on the computed volcano plot's descriptor (which, in turn, provides the TOF estimate, *vide infra*). These surrogate models allow us to bypass otherwise time-consuming experiments or computations and evaluate the fitness of new candidates generated during genetic optimization.

In the following sections, we describe in detail the individual components of the NaviCatGA pipeline (Fig. 1B), highlighting how they are adapted to find organocatalysts with a broad substrate scope. We then discuss the results of the evolutionary experiments, along with the chemical conclusions, in the Results and discussion section.

### Target property and reaction database

**Fig. 2:**
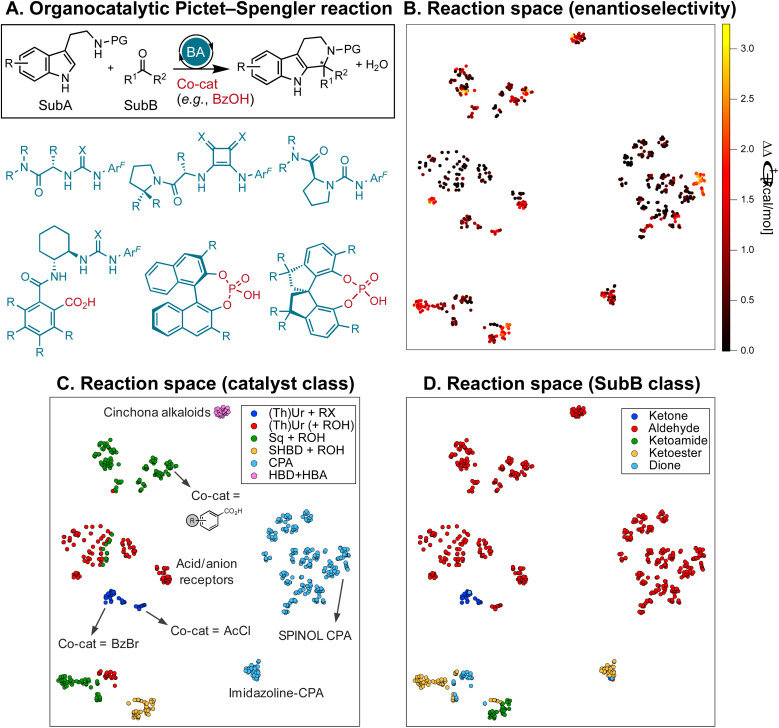
(A) Pictet–Spengler cyclization of tryptamine derivatives (SubA, PG = protecting group, H, or OH) and carbonyls (SubB) in the presence of chiral organocatalysts and weak acid co-catalysts. Examples of hydrogen-bond donors, acid/anion receptor catalysts, and chiral phosphoric acids are shown. Ar^F^ = 3,5-CF_3_-C_6_H_3_, X = O/S. (B)–(D) 2D t-SNE map^52^ of the reaction space on the basis of the concatenated MFPs of the substrates and catalysts color-coded by the experimental selectivity (ΔΔ*G*^‡^, B), catalyst class (C), and SubB class (D). (Th)Ur = (thio)ureas, Sq = squaramides, SHBD = single-hydrogen-bond donors, CPA = chiral phosphoric acids, HBA = hydrogen-bond acceptor, RX = benzoyl bromide or acyl chloride (BzBr, AcCl), ROH = carboxylic acid (*e.g.*, BzOH, AcOH).

The target of the inverse design strategy (Fig. 1B) is “generality” *i.e.*, high enantioselectivity and activity across a wide and diverse substrate scope. Inspired by recent work by Jacobsen *et al.*,^10^ we investigate the asymmetric Pictet–Spengler reaction^53–55^ of tryptamine derivatives and carbonyl compounds (Fig. 2A), one of the most important methods for the synthesis of privileged pharmacophores such as tetrahydro-β-carbolines, due to the diversity of catalyst chemotypes capable of inducing high enantioselectivity. Although dozens of systems have been reported,^56^ employing a variety of organocatalysts such as chiral phosphoric acids (CPAs)^57^ or single-^58^ and dual-hydrogen-bond donors (S/DHBD)^59^ used cooperatively with weak acids or bearing an acidic functional group internally,^60^ no method has found widespread application, since each study was focused on a limited number of substrates. This reaction constitutes an ideal case study to develop an optimization strategy with generality as primary target.^10^

At the onset of our investigation, we curated a database of 820 Pictet–Spengler condensations from the literature.^10,58,61–73^ For simplicity, we constrain ourselves to protected or unprotected tryptamines (as shown in Fig. 2A), excluding isotryptamines,^74^ aryl ethanols,^75,76^ phenethylamines,^77^ and other substrates involved in more complex cascade reactions.^78–85^ The database contains 240 unique transformations (*i.e.*, tetrahydro-β-carboline products) of 33 SubA and 164 SubB (aldehydes, ketones, α-ketoacids/esters/amides, and α-diones), catalyzed by 160 distinct organocatalysts and 30 co-catalysts (carboxylic acids, acyl and benzoyl chlorides and bromides). It is visualized in Fig. 2B with a 2D t-SNE map^52^ based on the concatenated Morgan FingerPrints^86,87^ (MFPs) of the catalyst, co-catalyst, and substrates, where each point representing a reaction is colored according to its selectivity (ΔΔ*G*^‡^ = −*RT* ln|e.r.|, with e.r. being the experimentally measured enantiomeric ratio). The map is divided into two regions, the right-hand side containing cyclizations catalyzed by CPAs, the left-hand side those with single and dual-HBDs (Fig. 2C); 75% of reactions involve aldehydes as SubB (top and middle parts of the map), while condensations of other carbonyl compounds are located in the lower regions (Fig. 2D).

**Fig. 3:**
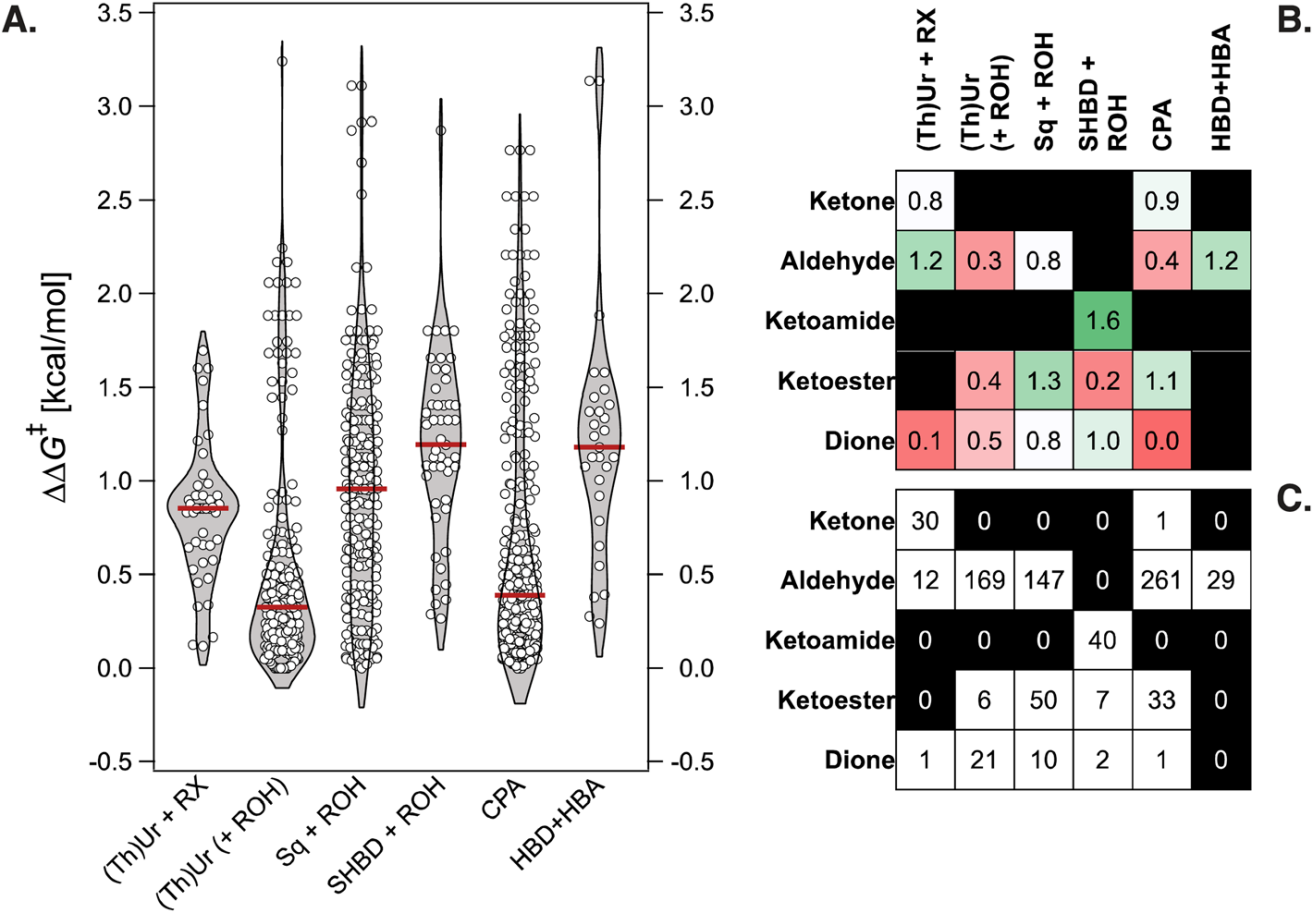
(A) Violin plots of experimental ΔΔ*G*^‡^ values in the literature database of 820 Pictet–Spengler reactions for six different classes of organocatalysts. The median is indicated with horizontal lines. RX = benzoyl bromide or acyl chloride (BzBr, AcCl), ROH = carboxylic acid (*e.g.*, BzOH, AcOH), HBA = hydrogen-bond acceptor. (B) Tabulated median ΔΔ*G*^‡^ values for different catalyst–substrate combinations from the literature database. (C) Tabulated number of reactions reported for different catalyst–substrate combinations from the literature database.

Despite “islands” of high enantioselectivity associated with catalysts being tested on a selected and limited class of carbonyl compounds (*e.g.*, SPINOL CPAs with aldehydes,^65^ or SHBDs with ketoamides,^58^*cf.*Fig. 2B–D), nearly 50% of the transformations display exceedingly low ΔΔ*G*^‡^ (<0.5 kcal mol^−1^, and 70% <1 kcal mol^−1^). The distribution of ΔΔ*G*^‡^ values for six families of organocatalysts [(thio)ureas with benzoyl bromide or acyl chloride co-catalyst, (thio)ureas, squaramides, or SHBD with carboxylic acid co-catalyst, CPAs, and bifunctional hydrogen-bond donor/acceptor cinchona alkaloids] is shown in Fig. 3A. Although certain chemotypes display high median ΔΔ*G*^‡^, choosing the catalyst for carrying out an enantioselective Pictet–Spengler reaction on a never-before-tested substrate simply based on literature precedence would lead to biased results, as only few catalyst–substrate combinations have actually been tested. This is emphasized in Fig. 3B and C, which display the median ΔΔ*G*^‡^ values for different substrate classes, along with the number of reactions reported. Finding general organocatalysts requires evaluating each candidate against a diverse panel of substrates, covering all types of tryptamine derivatives (SubA) and carbonyl compounds (SubB), which quickly becomes too expensive, supporting the need for predictive and generative models.

### Fitness function: evaluation of catalyst activity and selectivity

The database of experimental ΔΔ*G*^‡^ values (and the statistical model trained on it, *vide infra*) allows us to estimate the enantioselectivity of untested catalyst–substrates combinations. Regarding activity, we evaluate how close a catalyst's turnover is to the maximum achievable one using DFT computations and molecular volcano plots.^49–51^ Together, these measures of catalytic performance constitute the fitness function of the inverse design pipeline (Fig. 1B).

**Fig. 4:**
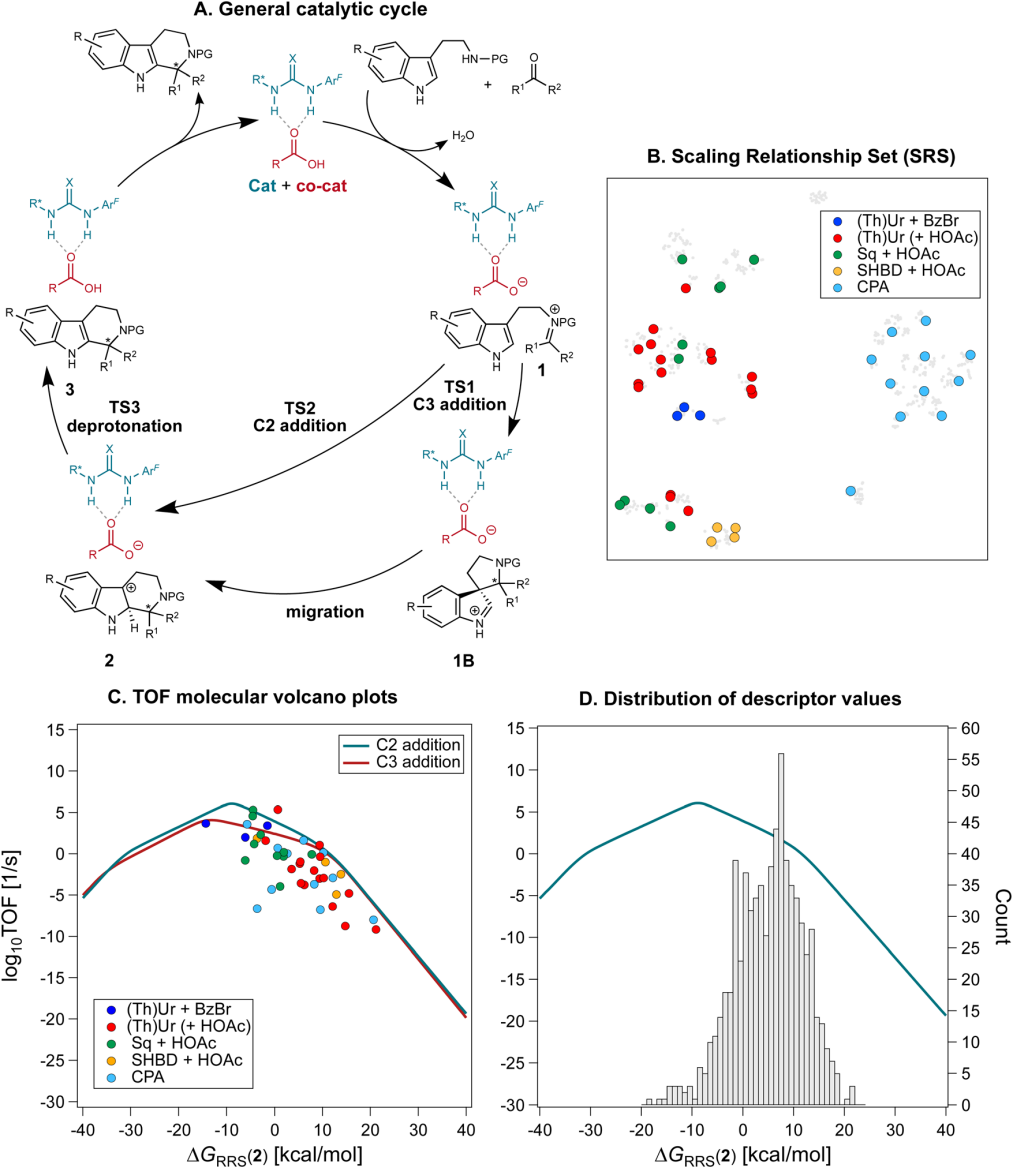
(A) General mechanism for the Pictet–Spengler reaction *via* anion-binding catalysis. (Thio)urea catalysts (X = O/S) with carboxylic acid co-catalysts are shown as an example. (B) The reactions used to construct molecular volcano plots (SRS) are plotted on the t-SNE map from Fig. 2, colored according to the nature of the organocatalyst. (C) Molecular volcano plots based on the C2 and C3 addition mechanism. The shaded areas denote the 95% confidence interval based on the Linear Free Energy Scaling Relationships. Computations were performed at the PCM(toluene)/M06-2X-D3/Def2-TZVP//M06-2X-D3/Def2-SVP level of theory. (D) Distribution of descriptor values and their location on the volcano plot.

Molecular volcanos provide a way of connecting a descriptor variable, typically the energy change associated with a step in a catalytic cycle (*x*-axis), to the overall catalytic performance (*y*-axis, expressed in terms of energy span or TOF),^49,88^ while simultaneously giving knowledge of the descriptor value corresponding to the volcano peak or plateau (maximum performance *i.e.*, the target for genetic optimization).^45^ Volcano plots are built from Linear Free Energy Scaling Relationships (LFESRs, Fig. S3†) that connect the value of the descriptor to the relative energies of the other cycle intermediates and transition states. While extensive details on how these plots are automatically constructed using the toolkit volcanic^51^ are given in the Computational details and elsewhere,^51^Fig. 4A shows the mechanism of the Pictet–Spengler reaction,^89^ whose knowledge is fundamental for building the volcanos. Following condensation of the β-arylethylamine (SubA) with the carbonyl compound (SubB) and formation of iminium ion 1, nucleophilic attack by the aryl group and cyclization can occur either directly at position C2 of the indole *via*TS2, or at C3 *via*TS1 to form the five-membered aza-spiroindolenine 1B, which undergoes C–C migration to yield 2. Deprotonation of 2 by the conjugate base of the acid co-catalyst, or of the CPA catalyst, is then necessary to form the tetrahydro-β-carboline product.

Constructing molecular volcanos requires computing the potential energy profiles of a medium-sized pool of sterically and electronically diverse systems.^51^ 44 reactions from the Pictet–Spengler database are selected *via* farthest point sampling of the 2D t-SNE map. This Scaling Relationships Set (SRS, Fig. 4B) comprises 39 unique transformations (*i.e.*, products) of 11 SubA and 31 SubB, catalyzed by 33 different organocatalysts. Because the mechanism must be the same for all systems investigated, reactions catalyzed by cinchona alkaloid HBD + HBA (corresponding to the pink cluster in the t-SNE map, Fig. 2C) are excluded, as these bifunctional catalysts have been shown to operate *via* a different mechanism.^67^ On the other hand, extensive mechanistic studies^68,89–93^ have demonstrated the viability of the mechanism shown in Fig. 4A for reactions catalyzed by (thio)urea HBDs, acid/anion receptors, and CPAs.

With the SRS, TOF molecular volcanos^49^ for concerted C2 and stepwise C3 addition are constructed automatically using volcanic^51^ and the relative energy of intermediate 2 as descriptor (Fig. 4C). Computations are performed at the PCM(toluene)/M06-2X-D3/Def2-TZVP//M06-2X-D3/Def2-SVP level of theory (see the Computational details); although exhaustive conformational sampling of each intermediate 2 is carried out with CREST,^94–96^ in order to reduce the computational cost only one conformer per stationary point on the Pictet–Spengler potential energy surface (PES) is used to construct the volcanos. The deviations of the points in Fig. 4C (each of which represents a Pictet–Spengler reaction) from the volcano curve may be attributed to differences in conformations between the various catalyst–substrate non-covalently bound complexes, which are characterized by a complex conformational landscape.

Mechanistic aspects of the Pictet–Spengler reaction, including the preferred pathway and the nature of the rate- and enantiodetermining step, have been a topic of intensive research:^97^ Jacobsen *et al.* found a strong energetic preference for C2 over C3 addition in reactions catalyzed by chiral thioureas,^89^ while You and co-workers showed that the spiroindolenine 1B acts as either a productive or non-productive intermediate depending on the shape of the PES.^93^ Evaluating the mechanism over a broad and diverse catalyst and substrate scope, as afforded by the SRS, reveals that, although the concerted pathways is generally preferred, the difference between the barriers for spiroindolization at C3 and electrophilic aromatic substitution at C2 is on average quite small (the volcanos are close to each other). Additionally, analysis of the LFESRs (Fig. S3†) shows that there is often not one single rate- and enantiodetermining step, as rearomatization *via* deprotonation (TS3) and C–C bond formation (TS1 or TS2) are almost isoenergetic: indeed, reactions are found for which TS2 and TS3 have similar degree of TOF-control^98^ (*i.e.*, the reaction rate is limited equally by C–C bond formation and deprotonation, see Fig. S4†). The location of the SRS on the volcano plots indicates that cyclizations of hydroxylamines in the presence of benzoyl bromide co-catalyst (blue points),^71^ as well as reactions of aldehydes catalyzed by squaramides (green points)^70^ display the highest TOFs. This observation is in line with the higher reactivity of ketonitrones^99^ and the stronger H-bonding ability of squaramides, which has been found to correlate with faster turnover.^100^ Conversely, the performance of CPAs and other DHBDs is strongly dependent on the nature of the substrates, as evinced by the bigger spread of TOF values. Among the poorest performing organocatalysts, sulfinamido urea derivatives^101^ and carboxylic acids equipped with anion-recognition sites^66^ are found lower on the volcano.

**Fig. 5:**
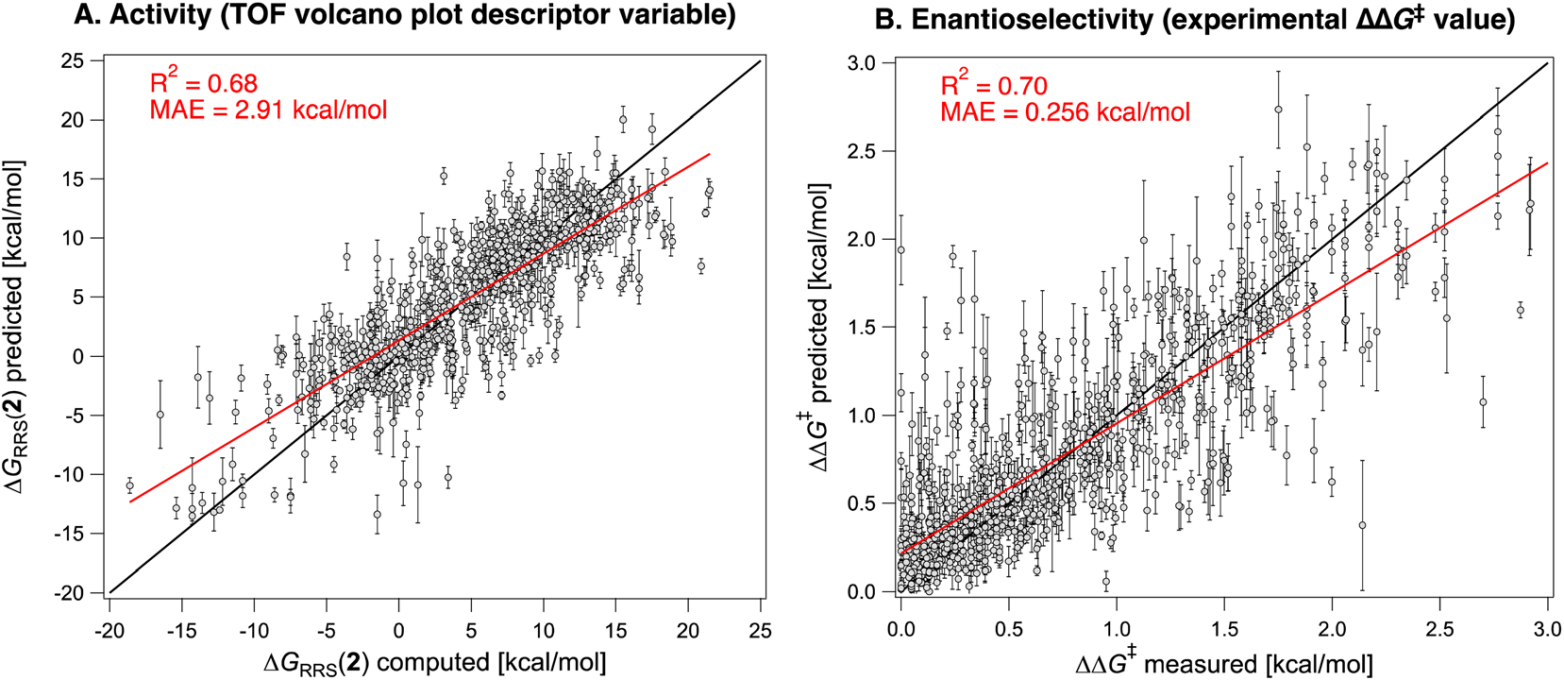
XGBoost models predicting the (A) descriptor variable [Δ*G*_RRS_(2)] of the TOF molecular volcano plots, computed at the PCM(toluene)/M06-2X-D3/Def2-TZVP//M06-2X-D3/Def2-SVP level, and (B) the experimentally measured enantioselectivity (expressed as ΔΔ*G*^‡^) of the Pictet–Spengler reactions from the literature. Predictions are obtained by averaging those from a cross-validation scheme with 100 different random 90/10 train/test splits (633/70 for A, 738/82 for B). The error bars are obtained from the standard deviations from the 100 different train/test splits.

Having constructed the volcano plots and established the identity of the descriptor variable, we compute Δ*G*_RRS_(2) for all the reactions in the Pictet–Spengler dataset (703 datapoints *i.e.*, excluding reactions catalyzed by cinchona alkaloids owing to their different mechanism and those where only the carboxylic acid co-catalyst is varied, since HOAc is used throughout, see the Computational details). Structures are generated and optimized according to the pipeline described in the Computational details. Fig. 4D shows the Gaussian-type distribution of Δ*G*_RRS_(2) superimposed on the TOF volcano for C2 addition, centered around 7 kcal mol^−1^. Most Pictet–Spengler reactions are found on the right slopes of the volcano (*i.e.*, weak-binding side), and their turnover is limited by iminium ion formation and deprotonation of the tetrahydro-β-carboline intermediate (or C–C bond formation). Overall, only few condensations have TOF close to the theoretical maximum. We then use this dataset to train a XGBoost machine learning model^102^ to predict Δ*G*_RRS_(2) using the concatenated Morgan FingerPrints of the substrates, catalyst, and co-catalyst (acetic acid, BzBr, or none) as reaction representation (Fig. 5A). A similar model is also trained on the whole Pictet–Spengler database (Fig. 2B*i.e.*, 820 datapoints, using the real identity of the carboxylic acid co-catalysts rather than acetic acid) to predict the experimental ΔΔ*G*^‡^ values. Despite the relatively large errors in Δ*G*_RRS_(2) predictions (MAE = 2.9 kcal mol^−1^) and for large ΔΔ*G*^‡^ values, these models are deemed to be an acceptable compromise between cost and accuracy and are used to accelerate fitness evaluation during genetic optimization (*vide infra*; see also Fig. S5† and 11 for out-of-sample predictions).^38^ The choice of the representation and regression method is dictated by the requirement of surrogate models used iteratively in generative molecular design to be fast and affordable. Although linear^103^ and non-linear^104^ models using stereoelectronic features^105^ (see Fig. S7† for multivariate linear regression analysis of the ΔΔ*G*^‡^ of reactions catalyzed by single- and dual-HBDs) or 3D structures as input^106,107^ have been extensively developed for reaction outcome prediction,^108^ they often depend on DFT computations of relatively expensive properties (*e.g.*, vibrational frequencies and intensities, polarizabilities)^109^ and are not adapted to the purpose of fast (GA) optimization, for which bypassing the DFT bottleneck is key.^38^ Conversely, 2D descriptors are typically much faster (and less susceptible to bias as they require less user input)^110^ and have been found to be cost-effective alternatives with good accuracy for experimental targets,^110–113^ sometimes even rivaling models using DFT features.^114^ The XGBoost model provides satisfying enantioselectivity predictions (MAE = 0.358 kcal mol^−1^, MSE = 0.221, Fig. S5†) on 46 out-of-sample reactions^115–117^ excluded from the original literature database, including condensations involving geminally-disubstituted tryptamines^117^ that are absent in the training set (Scheme S1†).

### Fragment database: the catalyst and substrate scope

The total combinatorial space explored during the evolutionary experiments is determined by the extent of the library of catalyst components and the scheme chosen to fragment them into building blocks. Here, we leverage the recently reported Organic Structures for CAtalysis Repository (OSCAR),^46^ which contains 4000 organocatalysts mined from the literature and CSD along with their corresponding molecular fragments. From OSCAR, we select 15 catalyst templates and 402 possible substituents (grouped into 4 categories R^1–4^ depending on which template they may substitute, see Tables S4 and S5† for a full list). The templates include 10 single- and dual-HBDs [(thio)ureas, (thio)squaramides, and prolyl-(thio)ureas] and 5 CPAs as shown in Fig. 2A (and Fig. S8†), which have been experimentally screened in the asymmetric Pictet–Spengler reaction. They are represented as flexible SMILES strings, written in such a way that different R^1–4^ can easily be introduced and exchanged, yielding valid SMILES. This results in a total combinatorial space of 2.85 × 10^8^ HBDs and 1428 CPAs. Note that only CPAs with equal substituents at the 6 and 6′ positions of the BINOL/SPINOL scaffold are considered: although this significantly reduces the size of their combinatorial space, it ensures synthetic accessibility, a common problem of generative models.^118^

**Fig. 6:**
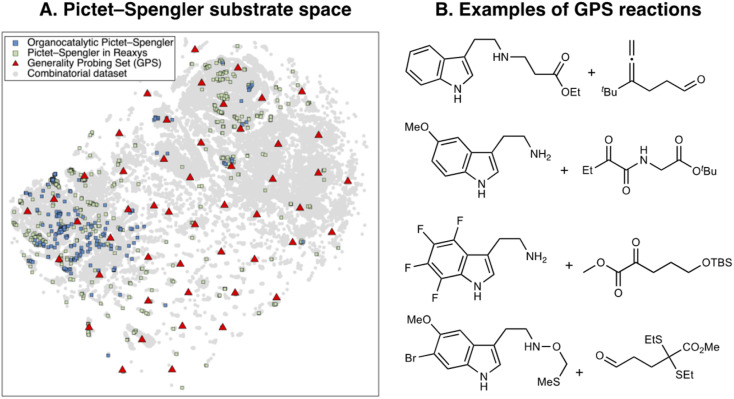
(A) 2D t-SNE map of the substrate scope on the basis of the concatenated MFPs of SubA and SubB. Blue squares indicate organocatalytic reactions, green squares reactions reported in Reaxys®, red triangles the Generality Probing Set (GPS) from this work. (B) Examples of reactions found in the GPS.

Having established the catalyst scope, we turn our attention to the substrate scope. Since our previous experiments with NaviCatGA were specificity-oriented,^45^ we implement a different workflow for selecting a representative subset of substrates for generality-driven genetic optimization. Inspired by recent work by Doyle *et al.*^119^ and Sigman *et al.*,^120,121^ we use the web platform Reaxys® to identify a list of 743 Pictet–Spengler reactions (selective and non-, catalytic and non-) between β-arylethylamines and carbonyl compounds. Additionally, 197 unprotected SubA, filtered according to molecular weight (<300 g mol^−1^), commercial availability, and functional group compatibility, are included. Combined with the 240 unique organocatalytic reactions from the original Pictet–Spengler database, we obtain 258 distinct tryptamine derivatives (SubA) and 379 carbonyls (SubB). The total combinatorial substrate space, shown in Fig. 6A, encompasses 97 782 possible tetrahydro-β-carboline products (grey circles).

Broadly speaking, examples from the literature (blue and green squares) cover the left half of the chemical space, which corresponds to unsubstituted tryptamines, while the right and bottom areas are sparsely covered. To generate a diverse and unbiased substrate scope for evolutionary experiments, we perform farthest point sampling and select 50 reactions aimed at covering the whole chemical space. Examples of this Generality Probing Set (GPS) are shown in Fig. 6B (the full list is given in Table S6†). Carbonyls (SubB) include predominantly aromatic and aliphatic aldehydes, as reflected by the popularity of these substrates in the Pictet–Spengler reaction (see also Fig. 3C),^10^ but also less explored α-diones, α-ketoamides, esters, and acids. Substituents on the tryptamine derivative (SubA) are present on all positions of the indole ring through mono-, di-, tri-, and even tetrasubstitution patterns, encompassing both electron-donating (*e.g.*, hydroxyl, methoxy, alkyl) and electron-withdrawing (*e.g.*, nitro, halide, ester) functional groups. This significantly contrasts the previously reported scope (*i.e.*, organocatalytic reactions from the literature or those mined from Reaxys®), dominated by monosubstituted β-arylethylamines. Approximately 60% of SubA in the GPS are unprotected, although a variety of protecting groups (*e.g.*, benzyl, 4-NO_2_-benzyl, methylthiomethyl ether,^122^ allyl^123^) are present.

## Results and discussion

### Evolutionary experiments

With the different components of the inverse design pipeline at hand (Fig. 1B), we perform evolutionary experiments using the NaviCatGA algorithm.^45^ Herein, we are trying to optimize multiple properties simultaneously: we are looking for general organocatalysts, meaning that they should exhibit high performance across the whole SubA–SubB substrate scope (represented by the GPS, Fig. 6), and we are looking for candidates with simultaneously high selectivity and activity.

**Fig. 7:**
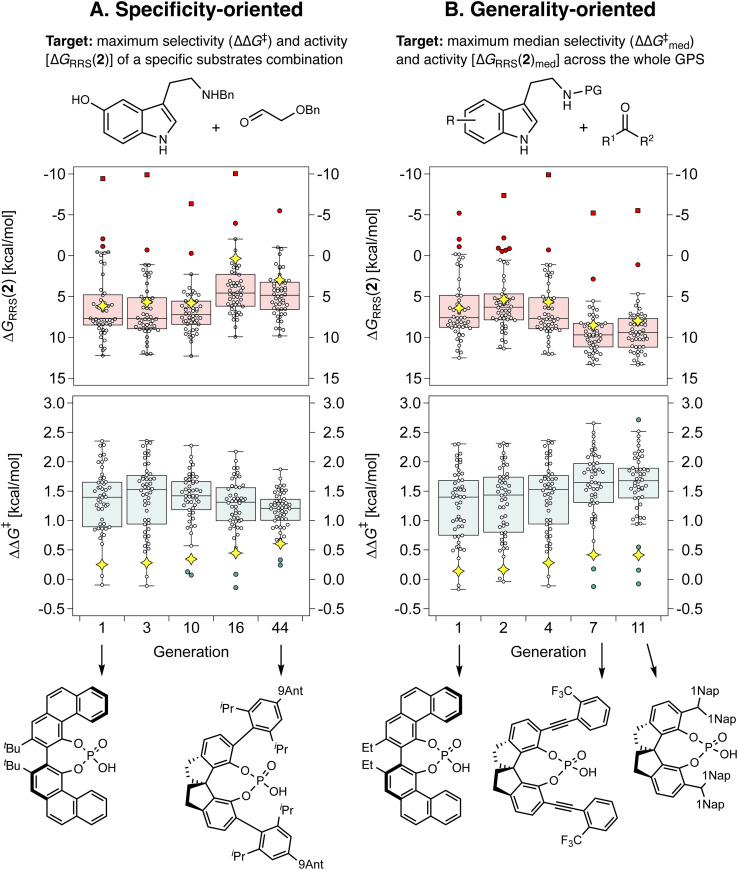
Box-and-whisker charts showing the evolution of ΔΔ*G*^‡^ and Δ*G*_RRS_(2) of the top individual in the CPA population for selected generations (*i.e.*, when the identity of the best-performing catalyst changes). Each datapoint corresponds to a reaction in the GPS, the yellow diamond indicates reaction 11 (shown in the top left). Outliers and far outliers are indicated with filled circles and squares, respectively. In (A), ΔΔ*G*^‡^ and Δ*G*_RRS_(2) of reaction 11 are optimized, whereas in (B) the median ΔΔ*G*^‡^ and ΔG_RRS_(2) of all reactions in the GPS are optimized.

To validate our strategy, we first compare specificity-oriented and generality-oriented optimization on the smaller CPA combinatorial space (*i.e.*, 1428 candidates; a similar experiment on the larger HBD space of 2.85 × 10^8^ possibilities is reported in Fig. S9†): in one case (Fig. 7A) the optimization targets are the selectivity (experimental ΔΔ*G*^‡^) and activity [Δ*G*_RRS_(2), the volcano plot descriptor] for the condensation of *N*_β_-benzylserotonin and benzyloxyacetaldehyde (reaction 11 in the GPS), predicted with the aforementioned XGBoost models. This particular combination of substrates was found to be associated with poor catalytic performance, and screening of all the 160 organocatalysts in the original literature dataset afforded median ΔΔ*G*^‡^ and Δ*G*_RRS_(2) of only 0.2 and 6.3 kcal mol^−1^, respectively. Note the volcano peak (maximum activity) corresponds to a Δ*G*_RRS_(2) value of −9.0 kcal mol^−1^. In the other case (Fig. 7B), we optimize the median ΔΔ*G*^‡^ and Δ*G*_RRS_(2) of all 50 reactions in the GPS. Given the multi-objective nature of each experiment (*i.e.*, simultaneous optimization of selectivity and activity), we scalarize^124^ the two targets seeking a minimum ΔΔ*G*^‡^ of 2.0 kcal mol^−1^, trying to reach a Δ*G*_RRS_(2) value of −9.0 kcal mol^−1^, but allowing activity to be marginally degraded if ΔΔ*G*^‡^ is increased (see the Computational details): this exemplifies a standard situation in which enantioselectivity is to be guaranteed and only subsequently turnover is to be optimized.

Fig. 7 depicts the results of the first set of experiments as box-and-whiskers charts, showing how ΔΔ*G*^‡^ and Δ*G*_RRS_(2) values are distributed across the GPS; only results for the best-performing catalyst in the population and only generations where the identity of the top candidate changes are shown. In the case of specificity-oriented optimization (Fig. 7A), ΔΔ*G*^‡^ of reaction 11 (yellow diamond) improves from 0.3 to 0.6 kcal mol^−1^ over the course of 44 generations; Δ*G*_RRS_(2) also improves from 6.1 kcal mol^−1^ to 3.0 kcal mol^−1^ (*i.e.*, approaching the volcano peak = −9.0 kcal mol^−1^, *cf.*Fig. 4C) but higher enantioselectivity comes at the expense of activity (*e.g.*, from generation 16 to 44). Although at the end of the experiment a SPINOL CPA is found with improved (albeit still relatively low) selectivity and good activity, the median ΔΔ*G*^‡^ decreases during the GA run, meaning that this organocatalyst is less general (conversely, this allows Δ*G*_RRS_(2)_med_ to actually improve, once again showing the conflicting nature of the two objectives).

In Fig. 7B, 
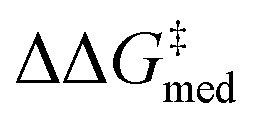
 increases from 1.4 in generation 1 to 1.5 kcal mol^−1^ in generation 4; activity also improves, with Δ*G*_RRS_(2)_med_ going from 7.6 to 6.3 kcal mol^−1^. To further enhance 
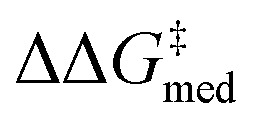
, NaviCatGA is forced to explore solutions in the activity-selectivity Pareto front with higher Δ*G*_RRS_(2)_med_ values (generation 7): this iteration corresponds to a change in catalyst scaffold, from VAPOL^125^ to SPINOL. In agreement with results from Jacobsen *et al.*,^10^ the SPINOL scaffold and 1-naphthyl substituents found in generation 11 are associated with good enantioselectivity across the GPS (
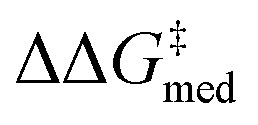
 = 1.7 kcal mol^−1^), as indicated by the smaller interquartile range (IQR, from 0.9 to 0.5 kcal mol^−1^). Therefore, even though ΔΔ*G*^‡^ for reaction 11 is lower than in the specificity-oriented optimization (0.4 kcal mol^−1^), a more general organocatalyst is discovered. Interestingly, the 2-CF_3_-phenylalkynyl substituent found in generation 7 was also identified by Denmark and co-workers as important for generality in the atroposelective disulfonimide-catalyzed iodination of 2-amino-6-arylpyridines,^26^ potentially suggesting that this group is also privileged across mechanistically-distinct reactions.^1^

**Fig. 8:**
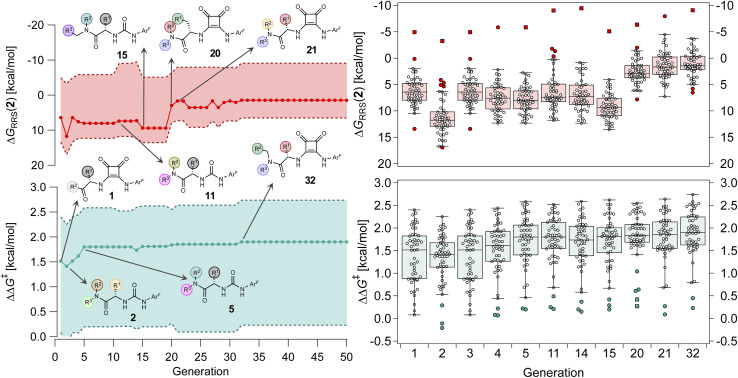
(Left) Evolution of ΔΔ*G*^‡^ and Δ*G*_RRS_(2) of the top individual in the HBD population over 50 generations. The solid lines indicate the median across the GPS, and the shaded areas represent the upper and lower values. Selected catalysts are shown, with different colored spheres representing different R^1–3^ substituents. (Right) Box-and-whisker chart of ΔΔ*G*^‡^ and Δ*G*_RRS_(2) for selected generations *i.e.*, only when the structure of the best-performing catalyst changes. Each datapoint corresponds to a reaction in the GPS. Outliers and far outliers are indicated with filled circles and squares, respectively.

**Fig. 9:**
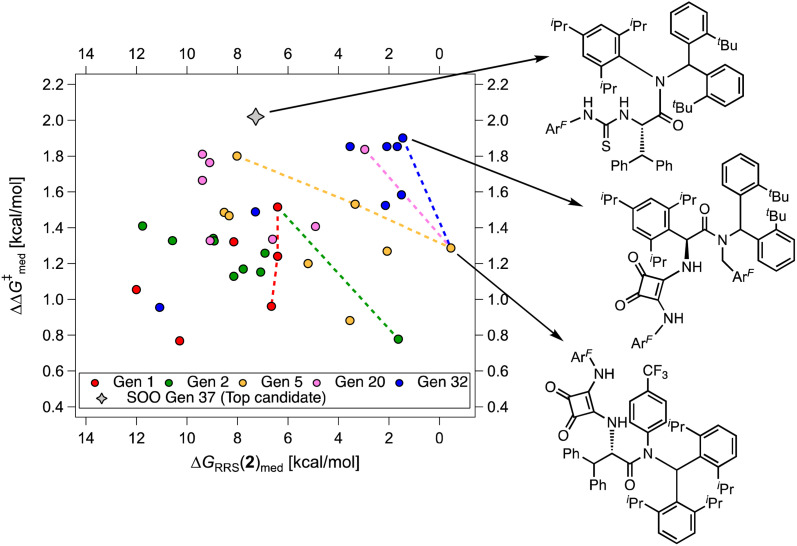
Median selectivity (
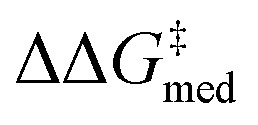
) *vs.* activity [Δ*G*_RRS_(2)_med_] scatter plot for multi-objective optimization on the HBD scope, color-coded by catalyst generation. The volcano peak (maximum activity) corresponds to Δ*G*_RRS_(2) = −9.0 kcal mol^−1^. The dashed lines show the connections for the set of “noninferior” solutions in the objective space (Pareto optimal solutions). The gray diamond represents the top candidate from the single-objective optimization experiment (SOO, generation 37).

Having validated the inverse design pipeline on the small CPA combinatorial space, we perform a second set of generality-oriented evolutionary experiments on the much larger HBD catalyst scope (2.85 × 10^8^ possible candidates). In the experiment reported in Fig. 8, the targets [
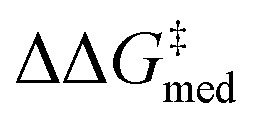
 and Δ*G*_RRS_(2)_med_] are scalarized as above, meaning we wish to optimize activity and selectivity simultaneously, but we allow turnover to be degraded in order to achieve higher enantioselectivities. Another GA run where only 
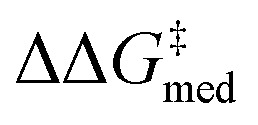
 is optimized (single-objective optimization, SOO) is shown in Fig. S10,† and results are discussed in the following section (the structure of the best-performing catalyst is shown in Fig. 9). Fig. S11† reports a third experiment where only Δ*G*_RRS_(2)_med_ is optimized, while in a fourth GA run (Fig. S12†) the two objectives (enantioselectivity and turnover) are scalarized differently *i.e.*, we allow 
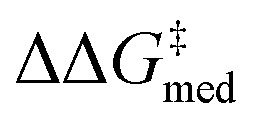
 to be marginally degraded in order to improve Δ*G*_RRS_(2)_med_ (see the ESI† for further details).

Over the first 5 generations, 
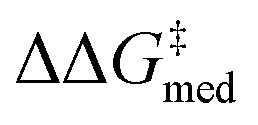
 increases from 1.5 kcal mol^−1^ to 1.8 kcal mol^−1^ while the IQR decreases, indicating that the top candidate is generally more selective across the GPS (Fig. 8). At the onset of the evolutionary experiment, NaviCatGA locates DHBDs with the amide-based template [–C(=O)NR_2_] as important for selectivity. Indeed, computational studies^89^ have shown that the amide O engages the substrate through an H-bonding interaction with the indoline N–H. This template^126^ is preserved throughout the GA run and preferred over catalysts containing the pyrrolidino-moiety:^1,127^ Jacobsen *et al.* similarly found that aryl pyrrolidine substituted thioureas had lower generality metric than acyclic amides in the Pictet–Spengler condensation of aldehydes.^10^ Regarding the identity of the hydrogen-bonding unit, for the first 20 generations ureas are selected over squaramides to increase 
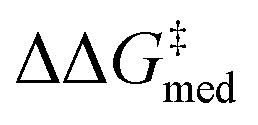
 but, in accordance with trends extracted from the volcano plots and the lower acidity/H-bonding ability of ureas *vs.* squaramides,^100,128^ this results in diminished activity [Δ*G*_RRS_(2)_med_ values farther away from the volcano peak of −9.0 kcal mol^−1^]. This situation exemplifies a typical problem in reaction optimization, where improving one objective is sometimes only possible at the expense of another.^129,130^ The same amino acid substituent (R^1^) is also maintained until generation 20, with NaviCatGA favoring the diphenyl group (black spheres in Fig. 8). At this particular iteration of the optimization, the squaramide HBD unit is “rediscovered”, which leads to a noticeable improvement in activity [Δ*G*_RRS_(2)_med_ from 9.4 to 3.0 kcal mol^−1^]. Although this is associated with only marginal increase in 
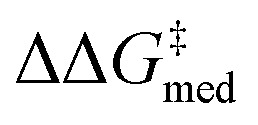
 (1.81 to 1.84 kcal mol^−1^), the IQR significantly decreases, and most reactions in the GPS have ΔΔ*G*^‡^ ≥ 1.7 kcal mol^−1^. Different R^1–3^ substituents are also selected, and in the remaining generations NaviCatGA explores different substitution patterns to achieve further activity and selectivity enhancements. In particular, Δ*G*_RRS_(2)_med_ is decreased to 1.5 kcal mol^−1^ with small IQR (generation 32), while 
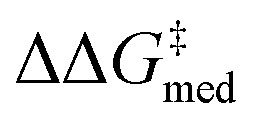
 reaches the value of 1.9 kcal mol^−1^. The most general organocatalyst found at the end of the evolutionary experiment exhibits the 2,4,6-^i^Pr-C_6_H_2_ substituent as R^1^, 3,5-CF_3_-C_6_H_3_ as R^2^, and the CH(2-^*t*^Bu-C_6_H_4_)_2_ group in place of R^3^. Clearly, bulky substituents are privileged in inducing high enantioselectivity and activity across the GPS.

While Fig. 8 focuses on the best catalyst in each generation, Fig. 9 shows how different individuals in a generation occupy the objective space. At each iteration of the NaviCatGA run, a number of solutions to the optimization problem exist, representing tradeoffs between the two objectives. Together, these catalysts constitute a set of nondominated optimal conditions, also known as Pareto front (dashed lines in Fig. 9).^129,131^ During the evolutionary experiment, the Pareto front moves towards higher 
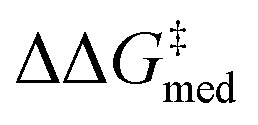
 and lower Δ*G*_RRS_(2)_med_ values (*i.e.*, closer to the volcano peak, −9.0 kcal mol^−1^), indicating an overall improvement in generality. The “ideal” organocatalyst *i.e.*, possessing the highest enantioselectivity and turnover possible over the whole substrate scope, would be located in the upper right corner of Fig. 9. The top catalyst from generation 32 constitutes the best compromise between selectivity and activity (
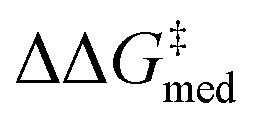
 = 1.9, Δ*G*_RRS_(2)_med_ = 1.5 kcal mol^−1^); conversely, nondominated points in the Pareto front of other generations represent candidates with higher activity but lower enantioselectivity (*e.g.*, generation 5, 
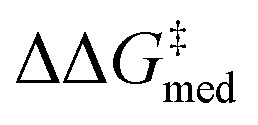
= 1.3, Δ*G*_RRS_(2)_med_ = −0.5 kcal mol^−1^). Therefore, the results of an evolutionary experiment may be used to identify catalysts that achieve different activity-selectivity tradeoffs, regardless of how the targets were initially scalarized. Fig. 9 also shows the top candidate from the single-objective optimization experiment (generation 37), which reaches higher 
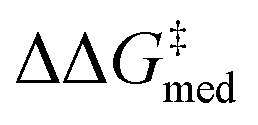
 (2.0 kcal mol^−1^) at the cost of significantly reduced activity [Δ*G*_RRS_(2)_med_ = 7.3 kcal mol^−1^]. In line with trends extracted from the volcano plot (Fig. 4C), the presence of the thiourea scaffold instead of the squaramide is associated with slower turnover,^100^ while the 2,4,6-^i^Pr-C_6_H_2_ and the CH(2-^*t*^Bu-C_6_H_4_)_2_ substituents ensure high enantioselectivity.

### Chemical insights into generality

**Fig. 10:**
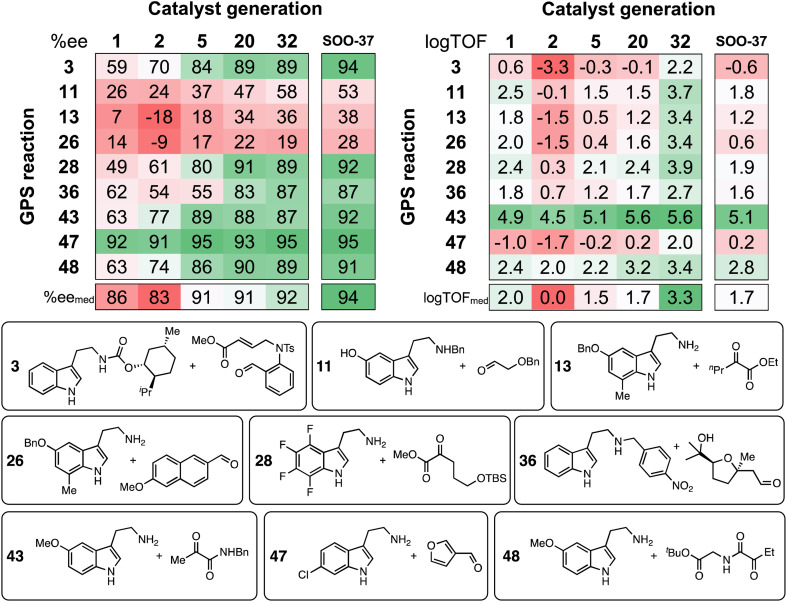
Calculated ee and log TOF values from the predicted ΔΔ*G*^‡^ and Δ*G*_RRS_(2), respectively. Results are shown for selected catalyst generations (*x*-axis) and reactions in the GPS (*y*-axis), while ee and log TOF median values (bottom) consider all 50 reactions in the GPS. SOO-37 is the top catalyst from the single-objective optimization experiment (structure shown in Fig. 9). Selected SubA and SubB combinations are shown.

Tabulation of the results of the evolutionary experiments on the HBD space as a heatmap, converted to ee and log TOF values (Fig. 10) shows that, although a catalyst with good median selectivity and activity is found (% ee_med_ = 92, log TOF_med_ = 3.3), some reactions in the GPS are always associated with poor performance *i.e.*, no matter how the structure of the catalyst evolves during the optimization, certain tetrahydro-β-carboline products may not be obtained in high ee or TOF. This is in contrast to the majority of condensations in the GPS, where selectivity and activity significantly improve as the structure of the organocatalyst is optimized. Reactions 28, 36, and 48 are included in Fig. 10 as examples: these transformations involve a variety of carbonyl compounds (α-ketoester, α-ketoamide, aldehyde) and electron-poor, neutral, and -rich indoles, showing that candidates with good generality across distinct substrate classes are indeed discovered. Note that, due to deviations in the LFESRs associated with the complex conformational space of the catalyst–substrate non-covalently bound complexes (Fig. 4C and S3†), significant differences between predicted and computed TOF values (up to several log units) may be expected.

Regarding the challenging areas of chemical space, the best-performing HBD organocatalyst from the multi-objective optimization experiment is predicted to achieve ee values of only 36% and 19% in reactions 13 and 26, respectively. Both condensations involve an unprotected β-arylethylamine (SubA) substituted at the 7-position of the indole ring; similarly, Suzuki and co-workers found that 7-methyltryptamine and ethyl 2-oxopentanoate could only be converted in 45% ee.^72^ These results can be explained in terms of steric effects of the methyl group on the substrate disrupting key non-covalent interactions between the catalyst's amide O and the indole N–H, which are evidently essential for inducing high enantioselectivity.^89^ The top candidate from the single-objective optimization (SOO-37) affords only marginal improvements for these substrate combinations (38% and 28% ee). Through the specificity-oriented optimization of reaction 13 (Fig. S13†), a urea-based organocatalyst with improved, albeit still low enantioselectivity (53% ee), slow turnover (log TOF = 0.7 s^−1^) and low generality is discovered, highlighting the limitation of an inverse design strategy based on the combinatorial exploration of known catalyst fragments on pre-described scaffolds.

Considering activity, throughout the NaviCatGA run reactions 3 and 47 are underperforming: according to the volcano plot (Fig. 4C), the formation of the corresponding protonated tetrahydro-β-carboline 2 is energetically unfavorable, in line with the electron-deficient nature of SubA and the electron-withdrawing character of the aldehyde substituent, which hinders the rate-determining deprotonation step. Regardless of the specific substitution patterns the GA may explore during the optimization, finding organocatalysts that non-covalently stabilize such unstable intermediates is clearly a challenge. Reaction 47 also exemplifies a situation where high selectivity and activity are incompatible: while most HBD organocatalysts explored during the evolutionary experiment are predicted to exhibit large ΔΔ*G*^‡^ values, the TOF always remains far from the theoretical maximum indicated by the volcano. Conversely, reaction 43, which features an electron-rich indole and an α-ketoamide (essentially an activated carbonyl compound),^132^ has predicted TOF always close to the volcano peak, while selectivity is more challenging to optimize,^58^ and ee values considerably improve during the GA run (from 63% to 87%).

**Fig. 11:**
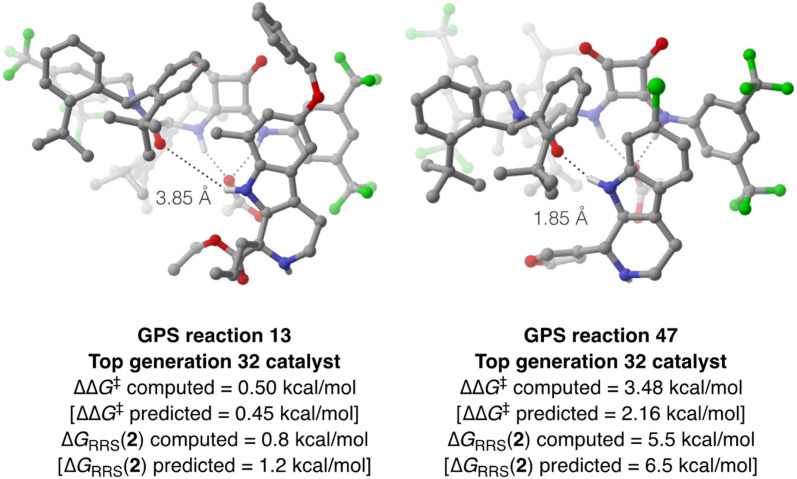
Energetically lowest-lying TS for the deprotonation/rearomatization step (TS3) of the tetrahydro-β-carboline intermediate of GPS reaction 13 (left) and 47 (right) with the top-performing organocatalyst from generation 32. The distance between the catalyst's amide O and the indole N–H is shown. Computed and predicted enantioselectivity (expressed in terms of ΔΔ*G*^‡^) and activity [expressed in terms of Δ*G*_RRS_(2)] values are reported.

To verify the accuracy of the ML predictions reported in Fig. 10 and probe the effect of a methyl substituent at the 7-position of the indole ring of SubA, DFT computations are performed on reactions 13 and 47 using the best organocatalyst from generation 32 in the multi-objective optimization (Fig. 11). Full conformational sampling of the two diastereomeric TSs for the rate- and enantiodetermining step (TS3) is carried out with CREST at the GFN2-xTB level, followed by optimization at the PCM(toluene)/M06-2X-D3/Def2-TZVP//M06-2X-D3/Def2-SVP level; enantioselectivity is computed based on the Gibbs free energy difference between the Boltzmann-weighted TSs conformers leading to the (*R*)- and (*S*)-tetrahydro-β-carboline products. Good agreement between the computed and predicted ΔΔ*G*^‡^ values is achieved for both reactions (Fig. 11); as expected from Fig. 5B, the XGBoost model underestimates the larger ΔΔ*G*^‡^ value of reaction 47, although such comparison must be taken with care since the XGBoost model is trained on experimental ΔΔ*G*^‡^'s, whereas Fig. 11 reports the results of DFT computations on TS3. Despite such limitation, this approach allows us to directly analyze the structure of the enantiodetermining transition states: as expected, the lowest-lying TS3 for reaction 13 features an elongated indole N–H⋯amide O intermolecular distance (3.85 Å), whereas a stronger hydrogen-bond is present in the catalyst–substrate complex of reaction 47 (1.85 Å). IRC computations^133^ are then performed to optimize the structure of intermediate 2 for both condensations, leading to relatively good agreement between computed and predicted Δ*G*_RRS_(2) values. The higher stability (*i.e.*, faster turnover according to the LFESRs and volcano plot) of the protonated tetrahydro-β-carboline 2 of reaction 13 is consistent with the electron-rich nature of the indole ring and the presence of an activated carbonyl compound such as ethyl 2-oxopentanoate, whereas the formation of 2 for reaction 47 is thermodynamically unfavorable [Δ*G*_RRS_(2) = 5.5 kcal mol^−1^] owing to the electron-poor character of the intermediate, which makes deprotonation slower.

Taken together, the results from the evolutionary experiment suggest that multiple “islands” of high ee or TOF exist in the catalyst–substrate chemical space, and that genetic optimization “expands” them. The discontinuity of the activity/selectivity-response surface is ultimately responsible for limiting generality;^134^ areas of poor performance are not simply due to structural aspects of the organocatalyst being mismatched to a particular substrates combination,^135^ but rather to the electronic character of a reaction intermediate inevitably leading to slow turnover or to the disruption of some key non-covalent interactions necessary for stereoinduction.

## Conclusions

Given the synthetic utility of catalytic methods that provide high enantioselectivities and activities across a wide assortment of substrates, we have developed an optimization workflow centered on the open-source genetic algorithm NaviCatGA^45^ and the OSCAR database^46^ with the aim of demonstrating how generative models^35^ are an enticing alternative to experimental^10^ or computational^34^ high-throughput screening, provided that the various component of the pipeline for *de novo* catalyst design are adapted to optimize generality as primary target. We have adopted a hybrid approach for scoring candidate organocatalysts that combines a mechanistic-guided strategy (*i.e.*, activity estimations through TOF molecular volcano plots^50^) with enantioselectivity predictions based on training on experimental data. Catalysts were generated from molecular building blocks extracted from OSCAR.^46^

We have tested our approach on the asymmetric Pictet–Spengler reaction^56^ because of the large amount of data available in the literature and the many catalyst chemotypes that have been tested on individual substrate classes, resulting in system-specific islands of high performance.^10^ We selected a broad and diverse substrate scope guided by mapping the chemical space of commercially and synthetically available tryptamine derivatives and carbonyl compounds tested in the Pictet–Spengler cyclization, and performed evolutionary experiments on this Generality Probing Set (GPS). Through multi-objective optimization, we have explored activity/selectivity trade-offs and located solutions in the Pareto front with good median performance. However, we found that even the top organocatalysts are underperforming in certain areas of substrate space, while other areas are less sensitive to the identity of the HBD/CPA catalyst. Analysis of these outliers provided support to hypotheses on the principle of stereoinduction^89^ and activity trends extracted from molecular volcanos, demonstrating how genetic optimization also yields mechanistic understanding and reveals structure–property relationships, as long as an unbiased substrate scope is chosen.^119^

Given these encouraging results, we believe the generality-oriented genetic optimization strategy we have introduced constitutes an efficient, cost-effective tool to probe large catalyst–substrate spaces and identify potential hits with a broad substrate scope, which may then be tested experimentally. The pipeline described herein is generalizable to any asymmetric reaction and can therefore help accelerate the discovery of general chiral catalysts for other transformations of interest.

## Computational details

### Electronic structure

The structure of both enantiomers of intermediate 2 in the catalytic cycle of the Pictet–Spengler reaction (Fig. 4A, labeled as “Big group pointing Up”, “BU”, or “Big group pointing Down”, “BD”, depending on the relative position of R^1^ and R^2^ in 2) were generated by substituting 3D fragments on 20 pre-optimized templates based on work by Jacobsen *et al.*^89^ using AaronTools^136,137^ and optimizing them with the semiempirical GFN2-xTB Hamiltonian^138^ in the gas phase. In analogy with computational studies by Jacobsen *et al.*,^89^ who found no clear trend relating the benzoic acid electronic properties to the reaction rate, the carboxylic acid co-catalyst, which sometimes contains large and bulky groups like triphenylmethyl, 9-anthracentyl, or 1-adamantyl,^70^ was modelled with acetic acid to simplify the conformational complexity and reduce the computational cost of the system. Conformational sampling of the resulting 703 complexes was carried out using the Conformer-Rotamer Ensemble Sampling Tool^94–96^ (CREST) at the GFN2-xTB//GFN-FF level of theory,^138^ constraining positions of the bond-forming atoms. The lowest-energy conformer was selected and optimized at the PCM(toluene)/M06-2X-D3/Def2-TZVP//M06-2X-D3/Def2-SVP level.^139–144^ The other intermediates and TSs in the SRS were located using scans and IRC computations.^133^ The PES of only one enantiomeric pathway (corresponding to “BD”-labeled structures) was generated to construct volcano plots (*vide infra*). Stationary points were characterized on the basis of their vibrational frequencies (minima with zero imaginary frequencies, TSs with one imaginary frequency). Thermal and entropic corrections were calculated using Grimme's quasi-RRHO approximation^145^ from frequencies computed at 298 K using the GoodVibes program^146^ with a frequency cut-off value of 100 wavenumbers. All DFT computations were carried out using Gaussian16 (revision C.01).^147^ The relative Gibbs free energies were automatically post-processed using the toolkit volcanic^51^ to establish LFESRs, determine the choice of the descriptor variable [the relative energy of intermediate 2, Δ*G*_RRS_(2)], and construct TOF–volcano plots. Extensive instructions on how volcano plots are constructed are given elsewhere,^51^ while the input for volcanic is provided in Table S1.†

### Statistical models

MFPs of catalysts, co-catalysts, substrates, and solvents with a fingerprint size of 1024 were generated using RDKit^148^ from their SMILES strings.^149^ Chemical space maps were generated using Scikit-learn^150^ on the basis of the concatenated MFPs with dimensions reduced to 100 using Principal Component Analysis, followed by t-SNE embedding^52^ with perplexity of 30 to further reduce the featurization to two dimensions for visualization. Random forest models from the XGBoost library were used with default hyperparameters. The input was the concatenated MPFs of Cat, Co-cat, SubA, SubB, and solvent for ΔΔ*G*^‡^, and of Cat, Co-Cat (*i.e.*, AcOH, BzBr, or none), SubA, and SubB for Δ*G*_RRS_(2). A cross-validation scheme was used with 100 different 90/10 training/test splits [738/82 for ΔΔ*G*^‡^, 633/70 for Δ*G*_RRS_(2)]. From the 100 different train/test splits, the target [ΔΔ*G*^‡^ or Δ*G*_RRS_(2)] was predicted approximately 10 times; these test predictions were then averaged to obtain one final prediction. The standard deviation from the test predictions were used to generate the error bars.^107^

### Evolutionary experiments

Genetic optimization was performed with the NaviCatGA algorithm.^45^ Genes were represented with SMILES strings (see Table S3† for a full list), and the assembler function generated the chromosomes by introducing the SMILES of different R^1–4^ substituents in a scaffold's SMILES string. The XGBoost models were used for fitness evaluation, with toluene fixed as solvent and benzoic (for ΔΔ*G*^‡^ evaluation) or acetic acid [for Δ*G*_RRS_(2) evaluation] fixed as co-catalyst; no co-catalyst was included in the GA runs on the CPA combinatorial space. Experiments were initiated with 10 randomized individuals per population, a mutation rate of 10%, a selection rate of 25%, and run for 50 generations. Multi-objective optimization was performed by integrating NaviCatGA with the achievement scalarizing function Chimera.^124^ Four objectives were hierarchically scalarized to obtain the final fitness value for each catalyst candidate *i*. The first objective was the median selectivity (
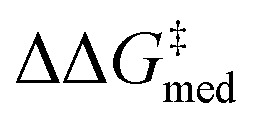
) across the GPS, which was required to be ≥2 kcal mol^−1^. Secondly, the activity of each candidate *i* was evaluated as 
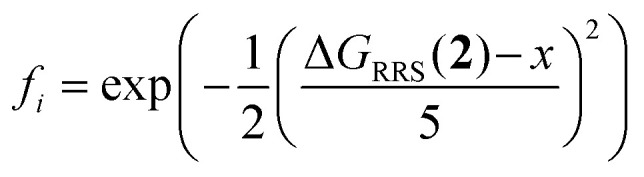
, a normalized Gaussian distribution centered on the target *x* (−9 kcal mol^−1^, the volcano peak); the median *f*_*i*_ value across the GPS was maximized with a 10% degradation threshold. The third and fourth objectives were the standard deviations of 
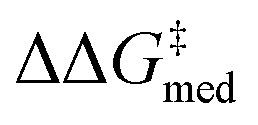
 and median *f*_*i*_ in the GPS, which were minimized with a 25% compromise.

## Data availability

Data can be found on the Materials Cloud (https://archive.materialscloud.org/record/2023.175). See the ESI† for further details.

## Author contributions

S. G. conceived the project, performed DFT computations, curated the data, and analyzed the results. P. v. G. trained the statistical models. R. L. designed and coded NaviCatGA and implemented it in the evolutionary experiments with help from S. G. L. B. helped curating the database of Pictet–Spengler reactions and generating 3D structures. A. M. ran preliminary computations initiating this work. S. G. wrote the manuscript with help and feedback from all authors. C. C. secured funding and provided supervision throughout.

## Conflicts of interest

There are no conflicts to declare.

## Supplementary Material

SC-015-D3SC06208B-s001
