# Supplementary material for: A genetic optimization strategy with generality in asymmetric organocatalysis as a primary target
Source: Chem Sci. 2024 Jan 31;15(10):3640–60. doi: 10.1039/d3sc06208b (PMC10915838; doi:10.1039/d3sc06208b)
Supplement: SC-015-D3SC06208B-s001 [file SC-015-D3SC06208B-s001.pdf]

Electronic Supplementary Material (ESI) for Chemical Science.
This journal is © The Royal Society of Chemistry 2024

Supporting Information for

A Genetic Optimization Strategy with Generality in Asymmetric Organocatalysis as Primary Target

Simone Gallarati,[a] Puck van Gerwen,[a,b] Ruben Laplaza,[a,b] Lucien Brey,[a] Alexander Makaveev,[a] and Clemence Corminboeuf[a,b,c,*]

[a]Laboratory for Computational Molecular Design, Institute of Chemical Sciences and Engineering, Ecole Polytechnique Fédérale de Lausanne (EPFL), 1015 Lausanne, Switzerland

[b]National Center for Competence in Research – Catalysis (NCCR-Catalysis), Ecole Polytechnique Fédérale de Lausanne (EPFL), 1015 Lausanne, Switzerland

[c]National Center for Computational Design and Discovery of Novel Materials (MARVEL), Ecole Polytechnique Fédérale de Lausanne (EPFL), 1015 Lausanne, Switzerland

*Email: clemence.corminboeuf@epfl.ch

Contents

1. Reaction Database S2
2. Linear Free Energy Scaling Relationships S3
3. Enantioselectivity Predictions S7
4. Fragments Database S10
5. Generality Probing Set S15
6. Evolutionary Experiments S16
7. Data Availability S22
8. References S23

1. Reaction Database

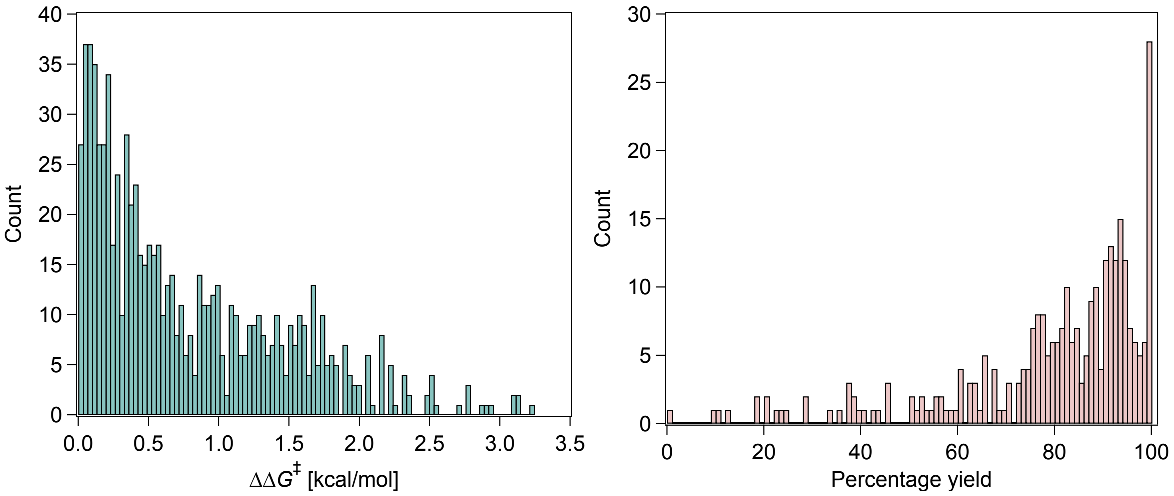

Figure S1. Distribution of experimental $\Delta\Delta G^{\ddagger}$ values (left) and experimental yields (right) in the literature database of 820 Pictet–Spengler reactions.

The histograms in Figure S1 show the distribution of experimental enantioselectivity and percentage yield values in the database of Pictet–Spengler condensations curated from the literature. Out of 820 reactions, only 36% (295) of yields were reported, predominantly for high-yielding reactions (60% of reported yields are > 80%). If we analyze the coverage of the reaction space by Pictet–Spengler condensations with reported yields (Figure S2), we see that large areas of chemical space are excluded from the database. For these reasons, DFT computations and molecular volcano plots[1] are used to estimate turnover frequencies and probe catalytic activity (*vide infra*).

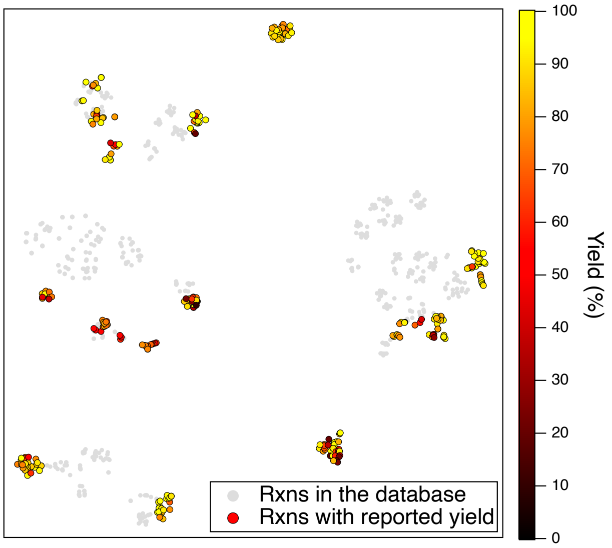

Figure S2. 2D t-SNE map[2] of the reaction space on the basis of the concatenated Morgan FingerPrints of the substrates, catalyst, and co-catalyst. Grey points represent all the reactions in the database (820), colored points (according to the experimental yield, 295) are the ones for which the yield is reported.

2. Linear Free Energy Scaling Relationships

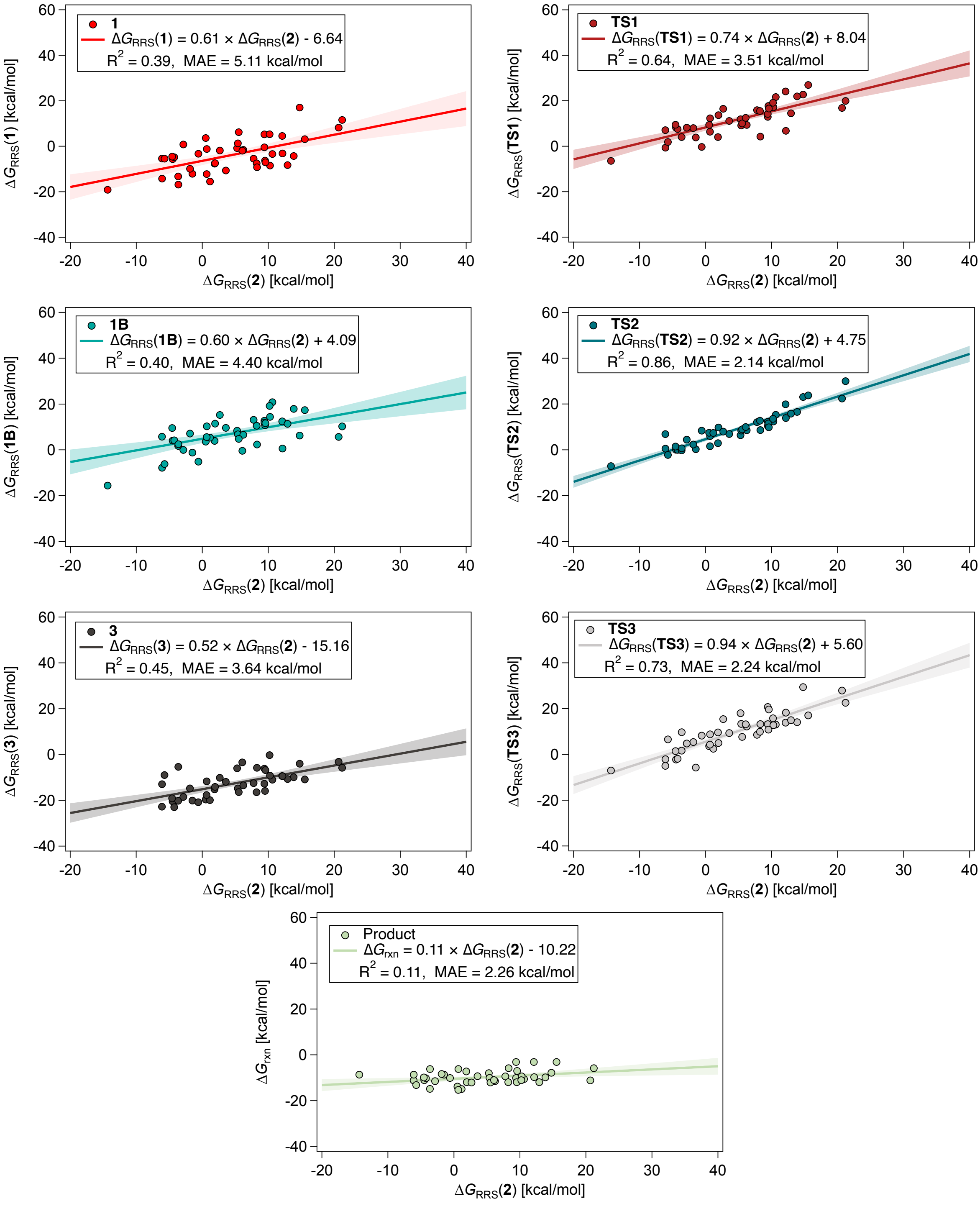

Figure S3. Linear Free Energy Scaling Relationships of catalytic cycle intermediates and TSs for the Scaling Relationship Set (only one enantiomeric pathway is considered). The *x*-axis is the chosen descriptor variable, $\Delta G_{RRS}(\mathbf{2})$. The shaded areas denote the 95% confidence intervals.

The TOF molecular volcano plots were constructed based on the LFESRs shown in Figure S3. Detailed instructions are provided elsewhere.[3] The open-source toolkit volcanic[3] was used to automatically generate the volcano plots. The input file for volcanic containing the relative Gibbs free energies of the

Scaling Relationship Set is provided in Table S1. $\Delta G_{RRS}(\mathbf{2})$ was identified by volcanic to be the best descriptor variable for constructing the molecular volcano plots for C2 (mean $R^2 = 0.57$, mean MAE = 2.76 kcal/mol, MAPE = 0.81) and C3 (mean $R^2 = 0.50$, mean MAE = 3.15 kcal/mol, MAPE = 1.35) addition. Figure S4 shows an exemplary potential energy surface (PES), highlighting the existence of transition states with similar degree of TOF control.[4] The location of the SRS reactions on the t-SNE plot of Figure 2 is reported in Table S2, along with the logTOF values for C2 addition and the experimental $\Delta\Delta G^{\ddagger}$.

Table S1. Relative Gibbs free energies (kcal/mol) of the Scaling Relationship Set. This is the format of the input file for volcanic.[3]

	Cat	Int1	TS1	Int1B	TS2	Int2	TS3	Int3	Product
**0_BD_Int2_I_Br**	0	-12.1	3.9	-1.2	0.3	-1.5	-5.7	-20.1	-8.6
**11_BD_Int2_I**	0	5.3	12.9	12.7	12.2	9.4	13.2	-12.7	-10.1
**11_BD_Int2_VII**	0	-7.6	4.0	3.9	2.9	1.9	9.6	-15.2	-7.2
**12_BD_Int2_X**	0	-5.4	7.1	5.8	6.9	-6.1	-5.0	-22.8	-11.1
**13_BD_Int2_X**	0	-5.7	8.1	4.0	0.0	-4.5	-2.2	-20.4	-11.1
**14_BD_Int2_III**	0	-0.8	11.9	8.4	6.4	5.3	18.0	-6.0	-8.0
**19_BD_Int2_I**	0	4.5	24.1	12.5	19.9	12.1	13.9	-9.4	-3.1
**1_BD_Int2_II_Br**	0	-19.1	-6.4	-15.6	-7.1	-14.3	-7.0	-22.6	-8.6
**1_BD_Int2_XII**	0	-3.4	21.7	20.8	15.3	10.6	13.1	-11.0	-10.5
**21_BD_Int2_III**	0	17.0	22.7	6.3	23.1	14.8	29.4	-4.0	-7.8
**25_BD_Int2_X**	0	-4.8	7.6	4.2	0.0	-4.3	-1.9	-23.0	-10.2
**27_BD_Int2_XII**	0	-13.3	4.0	1.7	-0.3	-3.6	1.6	-20.2	-14.8
**35_BD_Int2_VII**	0	-7.4	13.7	11.4	9.8	1.9	5.0	-14.1	-11.9
**36_BD_Int2_XII**	0	-4.3	21.9	17.9	16.5	13.9	14.1	-9.9	-9.8
**37_BD_Int2_X**	0	-4.5	9.5	9.5	1.4	-4.6	1.4	-19.1	-9.8
**3_BD_Int2_VI_Br**	0	-14.2	-0.6	-7.8	0.5	-6.1	-2.1	-12.9	-8.6
**3_BD_Int2_VIII**	0	-5.4	15.9	13.1	12.1	7.8	8.6	-12.5	-9.4
**42_BD_Int2_I**	0	1.3	9.1	6.8	8.4	5.4	13.3	-14.8	-10.0
**42_BD_Int2_XII**	0	-8.2	14.5	11.4	15.8	12.9	15.0	-10.7	-11.9
**45_BD_Int2_II**	0	-1.2	12.3	10.3	6.0	0.7	8.8	-11.7	-6.2
**4_BD_Int2_III**	0	-0.4	14.2	11.9	10.4	9.4	20.7	-6.2	-3.1
**4_BD_Int2_II**	0	-1.6	9.4	6.6	8.5	6.2	12.2	-13.5	-11.5
**4_BD_Int2_V**	0	3.1	26.9	17.4	23.8	15.5	17.0	-10.9	-3.1
**52_BD_Int2_II**	0	6.2	10.0	4.8	9.0	5.5	7.7	-16.4	-12.1
**55_BD_Int2_II**	0	-8.5	17.1	14.4	12.4	10.3	12.7	-9.2	-9.4
**59_BD_Int2_VII**	0	3.6	9.4	3.6	7.5	0.5	3.6	-19.7	-13.8
**61_BD_Int2_VII**	0	-15.5	22.9	5.3	7.4	1.2	2.5	-19.9	-14.9
**64_BD_Int2_I**	0	-9.9	8.0	7.1	2.3	-1.9	5.4	-14.8	-8.3
**69_BD_Int2_I**	0	-10.6	11.1	9.6	6.9	3.6	9.3	-11.9	-9.3
**6_BD_Int2_VII**	0	0.8	8.2	0.0	4.5	-2.9	4.8	-18.5	-11.4
**79_BD_Int2_I**	0	-6.3	17.7	10.8	11.5	9.5	10.8	-15.9	-11.9
**80_BD_Int2_I**	0	-7.5	15.5	10.6	12.7	8.2	9.9	-16.5	-11.9
**9_BD_Int2_V**	0	11.6	19.9	10.3	30.0	21.2	22.6	-5.8	-5.8
**0_BD_Int2_XIV**	0	-7.0	16.6	11.6	9.8	9.6	19.6	-6.8	-7.0
**100_BD_Int2_XIII**	0	-16.8	3.1	2.5	0.6	-3.6	9.7	-5.4	-6.2
**104_BD_Int2_XIII**	0	-3.3	-0.3	-5.1	8.4	-0.6	8.2	-20.8	-10.1
**10_BD_Int2_XIII**	0	-1.9	12.5	-0.4	9.9	6.1	13.2	-3.5	-11.0
**123_BD_Int2_XIII**	0	-5.4	1.9	-6.2	-2.2	-5.7	6.6	-8.9	-13.2
**29_BD_Int2_XVI**	0	-12.2	6.4	5.6	1.6	0.7	4.3	-17.7	-15.3
**31_BD_Int2_XVI**	0	-3.2	6.8	0.6	13.9	12.2	18.3	-9.5	-9.7
**6_BD_Int2_XVII**	0	5.3	19.1	19.2	13.5	10.2	15.8	-0.3	-11.1
**80_BD_Int2_XVI**	0	8.2	16.8	5.7	22.4	20.7	27.9	-3.2	-11.1
**85_BD_Int2_XVI**	0	-1.8	16.5	15.2	7.9	2.7	15.4	-10.2	-12.1
**94_BD_Int2_XVI**	0	-9.2	4.3	2.3	8.6	8.3	13.3	-5.8	-5.8

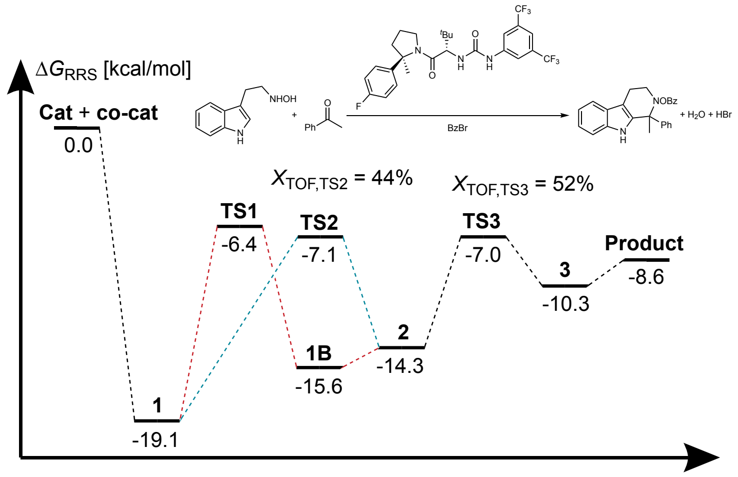

Figure S4. Exemplary PES of the Pictet–Spengler condensation of *N*-hydroxyl-tryptamine and acetophenone catalyzed by a urea Brønsted acid in the presence of benzoyl bromide co-catalyst (reaction labelled **1_BD_Int2_II_Br**).[5] Degrees of TOF control for **TS2** (44%) and **TS3** (52%) were computed using the energy span model.[4]

Table S2. Dimensions of the 2D t-SNE plot of the Pictet–Spengler database for the Scaling Relationships Set, along with the descriptor variable [ΔG_{RRS}(**2**), kcal/mol] and the logTOF values (1/s) for the C2 addition molecular volcano plot. The experimental $\Delta\Delta G^{\ddagger}$ value for these reactions is also reported.

Structure	Dim1	Dim2	ΔG_{RRS}(2)	logTOF	$\Delta\Delta G^{\ddagger}$
**0_BD_Int2_I_Br**	-15.16	-9.89	-1.5	3.4	0.90
**11_BD_Int2_I**	-20.06	2.44	9.4	1.1	0.50
**11_BD_Int2_VII**	-16.33	2.64	1.9	-0.3	0.44
**12_BD_Int2_X**	-7.87	21.11	-6.1	-0.8	0.07
**13_BD_Int2_X**	-7.54	21.52	-4.5	5.3	0.13
**14_BD_Int2_III**	-0.93	-6.34	5.3	-1.2	1.88
**19_BD_Int2_I**	-19.95	-0.85	12.1	-6.4	0.46
**1_BD_Int2_II_Br**	-16.65	-12.13	-14.3	3.7	0.53
**1_BD_Int2_XII**	-9.50	-44.79	10.6	-1.0	0.34
**21_BD_Int2_III**	-1.20	-5.20	14.8	-8.8	0.90
**25_BD_Int2_X**	-0.58	28.46	-4.3	1.2	2.53
**27_BD_Int2_XII**	-4.64	-43.73	-3.6	1.8	1.72
**35_BD_Int2_VII**	-22.33	-36.23	1.9	0.2	1.05
**36_BD_Int2_XII**	-4.43	-41.26	13.9	-2.5	2.17
**37_BD_Int2_X**	-15.59	28.57	-4.6	4.6	3.11
**3_BD_Int2_VI_Br**	-11.85	-12.01	-6.1	2.0	0.12
**3_BD_Int2_VIII**	-18.06	-40.82	7.8	-0.1	0.39
**42_BD_Int2_I**	-14.85	21.03	5.4	-1.0	0.55
**42_BD_Int2_XII**	-8.26	-41.13	12.9	-4.9	0.20
**45_BD_Int2_II**	-21.59	10.39	0.7	5.4	0.07
**4_BD_Int2_III**	-1.02	-1.92	9.4	-3.0	0.15
**4_BD_Int2_II**	-24.74	5.10	6.2	-3.8	0.19
**4_BD_Int2_V**	-9.44	2.38	15.5	-4.8	0.00
**52_BD_Int2_II**	-22.12	6.45	5.5	-3.6	0.07
**55_BD_Int2_II**	-14.33	-36.86	10.3	-2.9	0.63
**59_BD_Int2_VII**	-27.60	-34.23	0.5	-0.2	1.33
**61_BD_Int2_VII**	-28.65	-35.36	1.2	-4.0	1.60
**64_BD_Int2_I**	-24.76	-3.77	-1.9	1.6	2.06
**69_BD_Int2_I**	-24.74	-4.66	3.6	-1.9	3.24
**6_BD_Int2_VII**	-15.55	6.37	-2.9	2.3	0.15
**79_BD_Int2_I**	-18.02	-32.73	9.5	-0.4	0.00
**80_BD_Int2_I**	-18.10	-33.24	8.2	-2.0	0.34
**9_BD_Int2_V**	-9.65	4.23	21.2	-9.2	0.73
**0_BD_Int2_XIV**	38.69	-12.11	9.6	-6.8	0.71
**100_BD_Int2_XIII**	28.31	9.66	-3.6	-6.6	0.44
**104_BD_Int2_XIII**	35.02	-5.70	-0.6	-4.3	0.06
**10_BD_Int2_XIII**	30.06	-12.31	6.1	1.6	1.49
**123_BD_Int2_XIII**	19.66	-31.68	-5.7	3.6	1.53
**29_BD_Int2_XVI**	44.72	3.67	0.7	0.7	2.00
**31_BD_Int2_XVI**	35.16	3.28	12.2	-2.9	1.29
**6_BD_Int2_XVII**	24.01	2.47	10.2	0.2	0.17
**80_BD_Int2_XVI**	35.37	12.53	20.7	-8.0	0.19
**85_BD_Int2_XVI**	27.97	-3.71	2.7	0.0	0.23
**94_BD_Int2_XVI**	42.26	-3.05	8.3	-3.7	0.44

3. Enantioselectivity Predictions

3.1 Out-of-sample predictions

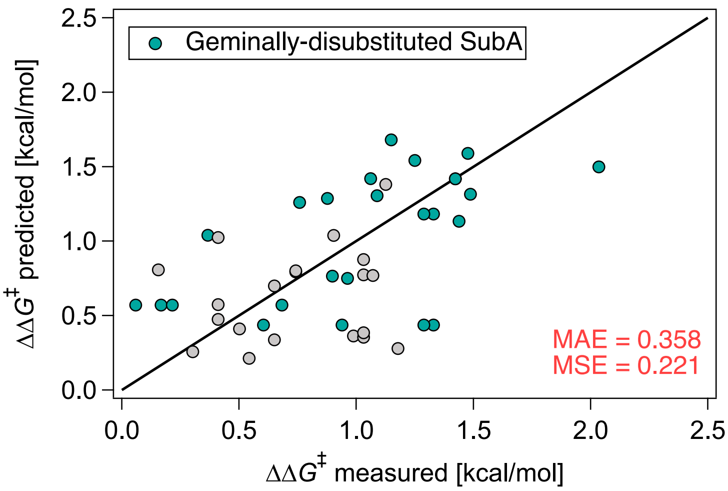

Figure S5. Out-of-sample enantioselectivity predictions on 46 reactions[6–8] withheld from the literature database using the trained XGBoost/MFP model. Teal points indicate reactions involving geminally-disubstituted tryptamines,[6] as shown in Scheme S1.

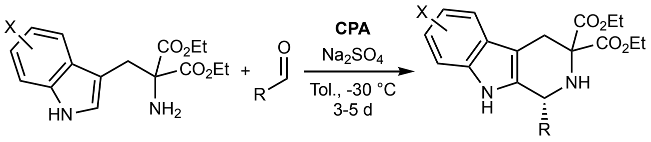

Scheme S1. CPA–catalyzed enantioselective Pictet–Spengler condensation of geminally-disubstituted tryptamines and aldehydes from List *et al.*[6]

3.2 Bayesian ridge regression of enantioselectivity

An alternative representation of the database of Pictet–Spengler condensations catalyzed by dual- and single-hydrogen-bond donors (S/DHBDs, 407 reactions) was constructed by extracting with MORFEUS[9] 119 global and local molecular features[10,11] from the corresponding catalytic cycle intermediate **2** ("BD"-labelled enantiomer, optimized at the PCM(Toluene)/M06-2X-D3/Def2-TZVP//M06-2X-D3/Def2-SVP level of theory as described in the Computational Details). Features include frontier molecular orbital energies, solvent accessible surface areas, polarizabilities, NBO charges, NMR chemical shifts, local nucleo/electrophilicities, bond distances, angles, dihedrals, and Sterimol parameters. FMO energies, NBO charges, NMR shifts, polarizabilities, and dipole moments were computed at the M06-2X-D3/Def2-TZVP level, whereas all other electronic descriptors were extracted with MORFEUS from computations at the GFN2-xTB level. Figure S6 shows the t-SNE plot of the featurized structures, indicating how reactions catalyzed by the same catalyst chemotype or involving the same substrate types are correctly grouped together.

We then used Bayesian ridge regression (BRR, a regularized variation of least-squares fitting) to parametrize a multivariate linear regression model[12] of the experimental $\Delta\Delta G^{\ddagger}$ values following our recently reported approach.[13] The resulting expression [equation (1)] contains 26 parameters extracted from either the protonated tetrahydro-*β*-carboline species (blue terms), the conjugated base of the co-catalyst (green term), or from the non-covalently bound Brønsted acid (red terms) (Scheme S2). Leave-one-out cross-validation (Figure S7) affords R^2 of 0.53 and mean absolute error of 0.34 kcal/mol. Despite these promising results, the cost associated with the optimization of structures generated during an evolutionary experiment and the computation of molecular features for untested catalyst–substrates combinations prohibit the use of this model for fitness evaluation during genetic optimization with NaviCatGA.

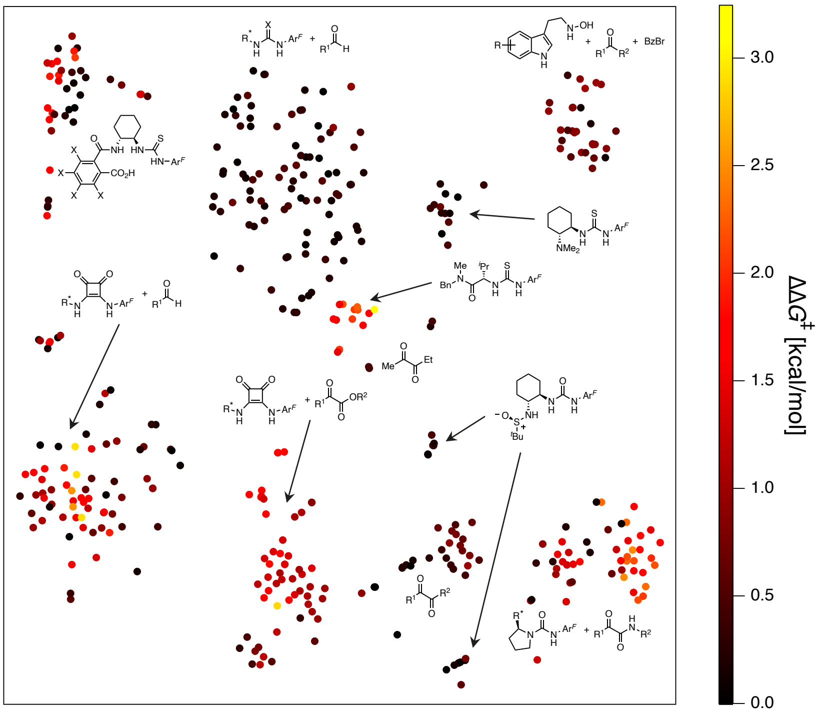

Figure S6. 2D t-SNE map[2] of the Pictet–Spengler reactions catalyzed by single- and dual-HBDs on the basis of the molecular (global and local) features of catalytic cycle intermediate **2**.

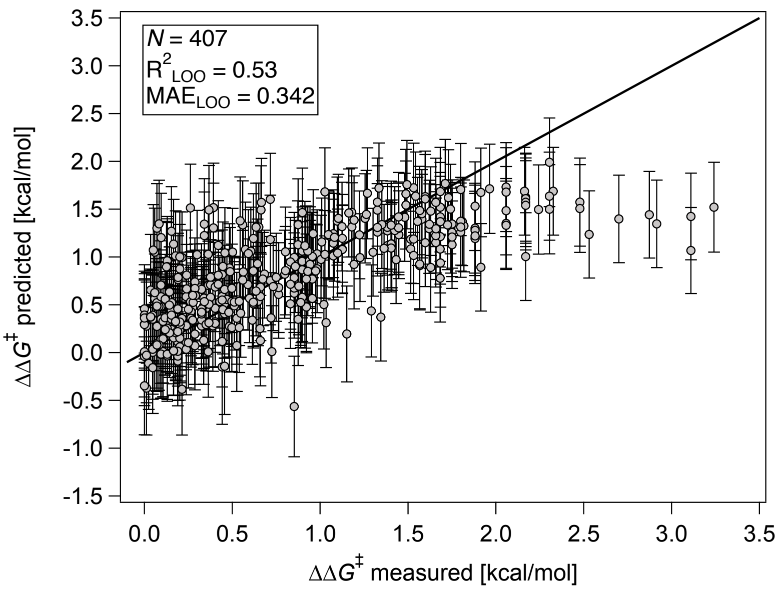

Figure S7. Multivariate linear regression of experimental $\Delta\Delta G^{\ddagger}$ values for Pictet–Spengler condensations catalyzed by single- and dual-HBDs (407 reactions). Bayesian ridge regression was used to parametrize the MLR models and provide uncertainty estimations (the error bars).[13]

(1) $$\Delta\Delta G^{\ddagger} = -0.10\boldsymbol{\mu} + 0.13\mathbf{V}_{bur[(C)H]} + 0.15\mathbf{P}_{int} - 0.13\mathbf{NBO}_{N(H)} - 0.16\mathbf{NBO}_{qC} - 0.81\mathbf{NBO}_{N(PG)} - 0.06\mathbf{NBO}_{C\text{-}HBD} - 0.17\mathbf{NBO}_{C1} - 0.13\mathbf{NBO}_{HBA} + 0.09\mathbf{SASA}_{(C)H} + 0.09\mathbf{NMR}_{qC} - 0.45\mathbf{NMR}_{N(PG)} + 0.09\mathbf{NMR}_{R4} + 0.18\mathbf{NMR}_{C2} - 0.28\boldsymbol{f}^{+}_{C+} - 0.08\boldsymbol{f}^{+}_{C(H)} + 0.08\boldsymbol{f}^{+}_{(C)H} + 0.16\boldsymbol{f}^{+}_{(N)H} + 0.19\boldsymbol{N}_{Avg(X)} - 0.10\boldsymbol{N}_{HBA} + 0.15\mathbf{d}_{HBA-(N)H} + 0.07\boldsymbol{\varphi}_{1(HNCX)} + 0.06\boldsymbol{\varphi}_{(HNNH)} + 0.07\mathbf{L}_{qC-R1} + 0.14\mathbf{B1}_{qC-R1} + 0.08\mathbf{B5}_{C1-R4} + 0.81$$

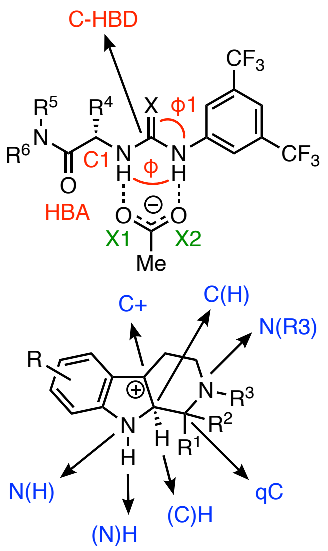

Scheme S2. Exemplary intermediate **2** for a Pictet–Spengler reaction catalyzed by a (thio)urea Brønsted acid and acetic acid co-catalyst with labels used in equation (1), where μ = global polarizability; V_{bur} = buried volume; P_{int} = universal quantitative dispersion coefficient;[14] SASA = solvent-accessible surface area; f^{+} = Fukui function for electrophilicity; N = local nucleophilicity; d = distance; φ = dihedral angle; L, B1, and B5 = Sterimol parameters.

4. Fragments Database

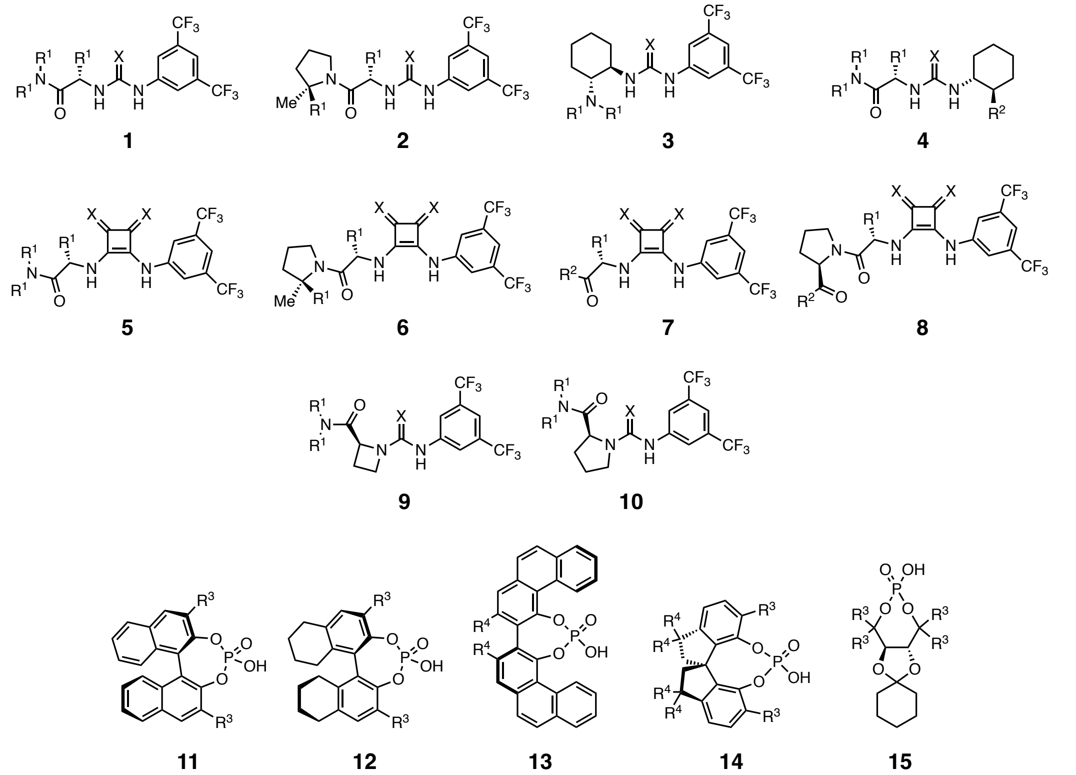

Figure S8. Database of Brønsted acid templates for evolutionary experiments. X = O/S.

Table S3. List of flexible SMILES strings of organocatalyst scaffolds for the evolutionary experiments.

#	SMILES
**1A**	O=C(N[C@H](C(N(R1)R1)=O)R1)NC1=CC(C(F)(F)F)=CC(C(F)(F)F)=C1
**1B**	S=C(N[C@H](C(N(R1)R1)=O)R1)NC1=CC(C(F)(F)F)=CC(C(F)(F)F)=C1
**2A**	O=C(NC1=CC(C(F)(F)F)=CC(C(F)(F)F)=C1)N[C@@H](R1)C(N2CCC[C@]2(C)R1)=O
**2B**	S=C(NC1=CC(C(F)(F)F)=CC(C(F)(F)F)=C1)N[C@@H](R1)C(N2CCC[C@]2(C)R1)=O
**3A**	O=C(N([C@H]5[C@H](N(R1)(R1))CCCC5))NC1=CC(C(F)(F)F)=CC(C(F)(F)F)=C1
**3B**	S=C(N([C@H]5[C@H](N(R1)(R1))CCCC5))NC1=CC(C(F)(F)F)=CC(C(F)(F)F)=C1
**4A**	O=C(N[C@H]5[C@H](R2)CCCC5)N[C@@H](R1)C(N(R1)R1)=O
**4B**	S=C(N[C@H]5[C@H](R2)CCCC5)N[C@@H](R1)C(N(R1)R1)=O
**5A**	O=C6C(N[C@H](C(N(R1)R1)=O)R1)=C(N(C1=CC(C(F)(F)F)=CC(C(F)(F)F)=C1))C6=O
**5B**	S=C6C(N[C@H](C(N(R1)R1)=O)R1)=C(N(C1=CC(C(F)(F)F)=CC(C(F)(F)F)=C1))C6=S
**6A**	O=C6C(N(C1=CC(C(F)(F)F)=CC(C(F)(F)F)=C1))=C(N[C@@H](R1)C(N2CCC[C@]2(C)R1)=O)C6=O
**6B**	S=C6C(N(C1=CC(C(F)(F)F)=CC(C(F)(F)F)=C1))=C(N[C@@H](R1)C(N2CCC[C@]2(C)R1)=O)C6=S
**7A**	O=C6C(N(C1=CC(C(F)(F)F)=CC(C(F)(F)F)=C1))=C(N[C@@H](R1)C(R2)=O)C6=O
**7B**	S=C6C(N(C1=CC(C(F)(F)F)=CC(C(F)(F)F)=C1))=C(N[C@@H](R1)C(R2)=O)C6=S
**8A**	O=C6C(N(C1=CC(C(F)(F)F)=CC(C(F)(F)F)=C1))=C(N[C@@H](R1)C(N2CCC[C@]2(C(R5)=O))=O)C6=O
**8B**	S=C6C(N(C1=CC(C(F)(F)F)=CC(C(F)(F)F)=C1))=C(N[C@@H](R1)C(N2CCC[C@]2(C(R5)=O))=O)C6=S
**9A**	O=C(NC1=CC(C(F)(F)F)=CC(C(F)(F)F)=C1)N5[C@H](C(N(R1)R1)=O)CC5
**9B**	S=C(NC1=CC(C(F)(F)F)=CC(C(F)(F)F)=C1)N5[C@H](C(N(R1)R1)=O)CC5
**10A**	O=C(NC1=CC(C(F)(F)F)=CC(C(F)(F)F)=C1)N5[C@H](C(N(R1)R1)=O)CCC5
**10B**	S=C(NC1=CC(C(F)(F)F)=CC(C(F)(F)F)=C1)N5[C@H](C(N(R1)R1)=O)CCC5
**11**	O=P6(O)OC7=C(R3)C=C(C=CC=C8)C8=[C@@]7[C@@]9=C(O6)C(R3)=CC%10=CC=CC=C9%10
**12**	O=P6(O)OC7=C(R3)C=C(CCCC8)C8=[C@@]7[C@@]9=C(O6)C(R3)=CC%10=C9CCCC%10
**13**	O=P6(O)OC7=C(C(C=CC=C8)=C8C=C9)C9=CC(R4)=C7C%10=C(O6)C%11=C(C=CC%12=C%11C=CC=C%12)C=C%10(R4)
**14**	O=P6(O)OC7=C([C@]8(C9=C(O6)C(R3)=CC=C9C(R4)(R4)C8)CC%10(R4)R4)C%10=CC=C7(R3)
**15**	O=P6(O)OC(R3)(R3)[C@H](O7)[C@H](C(R3)(R3)O6)OC%8%7CCCCC8

Table S4. List of SMILES strings of R^{1-2} substituents for HBD organocatalysts explored in the evolutionary experiments.

R^1	R^2
[H]	C
C	CC
CC	CCC
CCC	CCCC
CCCC	C(C)(C)
C(C)(C)	CC(C)(C)
CC(C)(C)	C(C)(CC)
C(C)(CC)	C(C)(C)(C)
C(C)(C)(C)	CC(C)(C)(C)
CC(C)(C)(C)	CC#C
CC#C	C1CCCC1
C1CCCC1	C1CCCCC1
C1CCCCC1	C1=CC=CC=C1
C1=CC=CC=C1	CC1=CC=CC=C1
CC1=CC=CC=C1	CCC1=CC=CC=C1
CCC1=CC=CC=C1	CC(C=C1)=CC=C1C2=CC=CC=C2
CC(C=C1)=CC=C1C2=CC=CC=C2	[C@H](C)C1=CC=CC=C1
[C@H](C)C1=CC=CC=C1	[C@H](C)C1CCCCC1
[C@H](C)C1CCCCC1	[C@H](C)C1=CC=CC2=C1C=CC=C2
[C@H](C)C1=CC=CC2=C1C=CC=C2	C(C1=CC=CC=C1)C1=CC=CC=C1
C(C1=CC=CC=C1)C1=CC=CC=C1	C(C1=CC=CC=C1C)C2=CC=CC=C2C
C(C1=CC=CC=C1C)C2=CC=CC=C2C	C(C1=CC=CC=C1CC)C2=CC=CC=C2CC
C(C1=CC=CC=C1CC)C2=CC=CC=C2CC	C(C1=CC=CC=C1C(C)C)C2=CC=CC=C2C(C)C
C(C1=CC=CC=C1C(C)C)C2=CC=CC=C2C(C)C	C(C1=CC=CC=C1C(C)(C)C)C2=CC=CC=C2C(C)(C)C
C(C1=CC=CC=C1C(C)(C)C)C2=CC=CC=C2C(C)(C)C	C(C1=C(C)C=CC=C1C)C2=C(C)C=CC=C2C
C(C1=C(C)C=CC=C1C)C2=C(C)C=CC=C2C	C(C1=C(CC)C=CC=C1CC)C2=C(CC)C=CC=C2CC
C(C1=C(CC)C=CC=C1CC)C2=C(CC)C=CC=C2CC	C(C1=C(C(C)C)C=CC=C1C(C)C)C2=C(C(C)C)C=CC=C2C(C)C
C(C1=C(C(C)C)C=CC=C1C(C)C)C2=C(C(C)C)C=CC=C2C(C)C	C(C1=C(F)C=CC=C1F)C2=C(F)C=CC=C2F
C(C1=C(F)C=CC=C1F)C2=C(F)C=CC=C2F	C(C1=C(OC)C=CC=C1OC)C2=C(OC)C=CC=C2OC
C(C1=C(OC)C=CC=C1OC)C2=C(OC)C=CC=C2OC	C(C1=CC(C)=CC(C)=C1)C2=CC(C)=CC(C)=C2
C(C1=CC(C)=CC(C)=C1)C2=CC(C)=CC(C)=C2	C(C1=CC(CC)=CC(CC)=C1)C2=CC(CC)=CC(CC)=C2
C(C1=CC(CC)=CC(CC)=C1)C2=CC(CC)=CC(CC)=C2	C(C1=CC(C(C)(C)C)=CC(C(C)(C)C)=C1)C2=CC(C(C)(C)C)=CC(C(C)(C)C)=C2
C(C1=CC(C(C)(C)C)=CC(C(C)(C)C)=C1)C2=CC(C(C)(C)C)=CC(C(C)(C)C)=C2	C(C1=CC(F)=CC(F)=C1)C2=CC(F)=CC(F)=C2
C(C1=CC(F)=CC(F)=C1)C2=CC(F)=CC(F)=C2	C(C1=CC(C(F)(F)F)=CC(C(F)(F)F)=C1)C2=CC(C(F)(F)F)=CC(C(F)(F)F)=C2
C(C1=CC(C(F)(F)F)=CC(C(F)(F)F)=C1)C2=CC(C(F)(F)F)=CC(C(F)(F)F)=C2	C(C1=CC(OC)=CC(OC)=C1)C2=CC(OC)=CC(OC)=C2
C(C1=CC(OC)=CC(OC)=C1)C2=CC(OC)=CC(OC)=C2	C(C1=CC(C=CC=C2)=C2C=C1)C3=CC(C=CC=C4)=C4C=C3
C(C1=CC(C=CC=C2)=C2C=C1)C3=CC(C=CC=C4)=C4C=C3	C(C1=CC=CC2=C1C=CC=C2)C3=CC=CC4=C3C=CC=C4
C(C1=CC=CC2=C1C=CC=C2)C3=CC=CC4=C3C=CC=C4	C1=CC=C(C)C=C1
C1=CC=C(C)C=C1	C1=CC=C(F)C=C1
C1=CC=C(F)C=C1	C1=CC=C(Cl)C=C1
C1=CC=C(Cl)C=C1	C1=CC=C(Br)C=C1
C1=CC=C(Br)C=C1	C1=CC=C(C#N)C=C1
C1=CC=C(C#N)C=C1	C1=CC=C(OC)C=C1
C1=CC=C(OC)C=C1	C1=CC=C(O)C=C1
C1=CC=C(O)C=C1	C1=CC=C([N+](=O)([O-]))C=C1
C1=CC=C([N+](=O)([O-]))C=C1	C1=CC=C(C(F)(F)(F))C=C1
C1=CC=C(C(F)(F)(F))C=C1	C1=CC=C(C(C)(C)(C))C=C1
C1=CC=C(C(C)(C)(C))C=C1	CC1=CC=C(C)C=C1
CC1=CC=C(C)C=C1	CC1=CC=C(F)C=C1
CC1=CC=C(F)C=C1	CC1=CC=C(Cl)C=C1
CC1=CC=C(Cl)C=C1	CC1=CC=C(Br)C=C1
CC1=CC=C(Br)C=C1	CC1=CC=C(C#N)C=C1
CC1=CC=C(C#N)C=C1	CC1=CC=C(OC)C=C1
CC1=CC=C(OC)C=C1	CC1=CC=C(O)C=C1
CC1=CC=C(O)C=C1	CC1=CC=C([N+](=O)([O-]))C=C1
CC1=CC=C([N+](=O)([O-]))C=C1	CC1=CC=C(C(F)(F)(F))C=C1
CC1=CC=C(C(F)(F)(F))C=C1	CC1=CC=C(C(C)(C)(C))C=C1
CC1=CC=C(C(C)(C)(C))C=C1	C1=CC(C)=CC(C)=C1
C1=CC(C)=CC(C)=C1	C1=CC(F)=CC(F)=C1
C1=CC(F)=CC(F)=C1	C1=CC(Cl)=CC(Cl)=C1
C1=CC(Cl)=CC(Cl)=C1	C1=CC([N+]([O-])(=O))=CC([N+]([O-])(=O))=C1
C1=CC([N+]([O-])(=O))=CC([N+]([O-])(=O))=C1	C1=CC(C(F)(F)(F))=CC(C(F)(F)(F))=C1
C1=CC(C(F)(F)(F))=CC(C(F)(F)(F))=C1	C1=CC(OC)=CC(OC)=C1
C1=CC(OC)=CC(OC)=C1	C1=CC(C(C)(C)(C))=CC(C(C)(C)(C))=C1
C1=CC(C(C)(C)(C))=CC(C(C)(C)(C))=C1	C1=C(C)C=CC=C1(C)
C1=C(C)C=CC=C1(C)	C1=C(C)C=C(C)C=C1(C)
C1=C(C)C=C(C)C=C1(C)	C1=C(C(C)(C))C=C(C(C)(C))C=C1(C(C)(C))
C1=C(C(C)(C))C=C(C(C)(C))C=C1(C(C)(C))	C1=C(Cl)C=C(Cl)C=C1(Cl)
C1=C(Cl)C=C(Cl)C=C1(Cl)	C1=C(C)C=C(C(C)(C)(C))C=C1(C)
C1=C(C)C=C(C(C)(C)(C))C=C1(C)	C1=CC(F)=C(F)C(F)=C1

C1=CC(F)=C(F)C(F)=C1	C1=C(F)C=CC=C1(F)
C1=C(F)C=CC=C1(F)	C1=C(C)C(C)=C(C)C(C)=C1(C)
C1=C(C)C(C)=C(C)C(C)=C1(C)	C1=C(F)C(F)=C(F)C(F)=C1(F)
C1=C(F)C(F)=C(F)C(F)=C1(F)	CC1=CC(C)=CC(C)=C1
CC1=CC(C)=CC(C)=C1	CC1=CC(F)=CC(F)=C1
CC1=CC(F)=CC(F)=C1	CC1=CC(Cl)=CC(Cl)=C1
CC1=CC(Cl)=CC(Cl)=C1	CC1=CC([N+]([O-])(=O))=CC([N+]([O-])(=O))=C1
CC1=CC([N+]([O-])(=O))=CC([N+]([O-])(=O))=C1	CC1=CC(C(F)(F)(F))=CC(C(F)(F)(F))=C1
CC1=CC(C(F)(F)(F))=CC(C(F)(F)(F))=C1	CC1=CC(OC)=CC(OC)=C1
CC1=CC(OC)=CC(OC)=C1	CC1=CC(C(C)(C)(C))=CC(C(C)(C)(C))=C1
CC1=CC(C(C)(C)(C))=CC(C(C)(C)(C))=C1	CC1=C(C)C=CC=C1(C)
CC1=C(C)C=CC=C1(C)	CC1=C(F)C=CC=C1(F)
CC1=C(F)C=CC=C1(F)	CC1=C(C)C=C(C)C=C1(C)
CC1=C(C)C=C(C)C=C1(C)	CC1=C(C(C)(C))C=C(C(C)(C))C=C1(C(C)(C))
CC1=C(C(C)(C))C=C(C(C)(C))C=C1(C(C)(C))	CC1=C(Cl)C=C(Cl)C=C1(Cl)
CC1=C(Cl)C=C(Cl)C=C1(Cl)	CC1=C(C)C=C(C(C)(C)(C))C=C1(C)
CC1=C(C)C=C(C(C)(C)(C))C=C1(C)	CC1=CC(F)=C(F)C(F)=C1
CC1=CC(F)=C(F)C(F)=C1	CC1=C(C)C(C)=C(C)C(C)=C1(C)
CC1=C(C)C(C)=C(C)C(C)=C1(C)	CC1=C(F)C(F)=C(F)C(F)=C1(F)
CC1=C(F)C(F)=C(F)C(F)=C1(F)	C1=CC=CC2=C1C=CC=C2
C1=CC=CC2=C1C=CC=C2	C1=CC=C(C=CC=C2)C2=C1
C1=CC=C(C=CC=C2)C2=C1	CC1=CC=CC2=C1C=CC=C2
CC1=CC=CC2=C1C=CC=C2	CC1=CC=C(C=CC=C2)C2=C1
CC1=CC=C(C=CC=C2)C2=C1	CN(C)CC1=CC=CC=C1
CN(C)CC1=CC=CC=C1	C1=CC2=C(C=CC=C2)C3=CC=CC=C31
C1=CC2=C(C=CC=C2)C3=CC=CC=C31	C1=C(C=CC=C2)C2=CC3=C1C=CC=C3
C1=C(C=CC=C2)C2=CC3=C1C=CC=C3	CC1=C(C=CC=C2)C2=CC3=C1C=CC=C3
CC1=C(C=CC=C2)C2=CC3=C1C=CC=C3	C1=C2C3=C(C=CC4=CC=CC(C=C2)=C43)C=C1
C1=C2C3=C(C=CC4=CC=CC(C=C2)=C43)C=C1	CC1=C2C3=C(C=CC4=CC=CC(C=C2)=C43)C=C1
CC1=C2C3=C(C=CC4=CC=CC(C=C2)=C43)C=C1	C(C1=CC=CC=C1)(C2=CC=CC=C2)O
C(C1=CC=CC=C1)(C2=CC=CC=C2)O	N
	N(C)
	N(CC)
	N(CCC)
	N(CCCC)
	N(C1=CC=CC=C1)
	N(CC1=CC=CC=C1)
	N(C)(C)
	N(CC)(CC)
	N(CCC)(CCC)
	N(CCCC)(CCCC)
	N(C)(C1=CC=CC=C1)
	N(C)(CC1=CC=CC=C1)
	N(C)(C1=CC=C(C)C=C1)
	N(C)(C1=CC=C(F)C=C1)
	N(C)(C1=CC=C(OC)C=C1)
	N(C)(C1=CC=C(C(F)(F)(F))C=C1)
	N(C)(C1=CC(C)=CC(C)=C1)
	N(C)(C1=CC(F)=CC(F)=C1)
	N(C)(C1=CC(Cl)=CC(Cl)=C1)
	N(C)(C1=CC([N+]([O-])(=O))=CC([N+]([O-])(=O))=C1)
	N(C)(C1=CC(C(F)(F)(F))=CC(C(F)(F)(F))=C1)
	N(C)(C1=CC(OC)=CC(OC)=C1)
	N(C)(C1=CC(C(C)(C)(C))=CC(C(C)(C)(C))=C1)
	N1C=CC=C1
	N1C(C)=CC=C1C
	N1C(C)=CC=C1(C1=CC=CC=C1)
	N1C(C2=CC=CC=C2)=CC=C1C3=CC=CC=C3
	N1C(C)=CC=C1(C1=CC=CC2=C1C=CC=C2)
	N1C(C)=CC=C1(C1=CC=C(C=CC=C2)C2=C1)
	N1C(C)=CC(C(OCC)=O)=C1(C1=CC=C(C=CC=C2)C2=C1)
	N1CCC2=C1C=CC=C2
	N1CC(C=CC=C2)=C2C1
	N1CCC2=C1C=C(OC)C=C2
	N1CC(C=CC(OC)=C2)=C2C1
	N1CCC2=C1C=C(Cl)C=C2
	N1CC(C=CC(Cl)=C2)=C2C1
	N1CC2=C3C1=CC=CC3=CC=C2

Table S5. List of SMILES strings of R^{3-4} substituents for CPA organocatalysts explored in the evolutionary experiments.

**R^3**	**R^4**
[H]	[H]
C	C
CC	CC
CCC	C(C)(C)
CCCC	C(C)(C)(C)
C(C)(C)	C1=CC=CC=C1
CC(C)(C)	
C(C)(CC)	
C(C)(C)(C)	
CC(C)(C)(C)	
C#C	
CC#C	
C#N	
F	
Cl	
Br	
C1CCCCC1	
C1=CC=CC=C1	
CC1=CC=CC=C1	
CCC1=CC=CC=C1	
CC(C=C1)=CC=C1C2=CC=CC=C2	
[C@H](C)C1=CC=CC=C1	
[C@H](C)C1CCCCC1	
[C@H](C)C1=CC=CC2=C1C=CC=C2	
C(C1=CC=CC=C1)C1=CC=CC=C1	
C(C1=CC=CC=C1C)C2=CC=CC=C2C	
C(C1=CC=CC=C1CC)C2=CC=CC=C2CC	
C(C1=CC=CC=C1C(C)C)C2=CC=CC=C2C(C)C	
C(C1=CC=CC=C1C(C)(C)C)C2=CC=CC=C2C(C)(C)C	
C(C1=C(C)C=CC=C1C)C2=C(C)C=CC=C2C	
C(C1=C(CC)C=CC=C1CC)C2=C(CC)C=CC=C2CC	
C(C1=C(C(C)C)C=CC=C1C(C)C)C2=C(C(C)C)C=CC=C2C(C)C	
C(C1=C(F)C=CC=C1F)C2=C(F)C=CC=C2F	
C(C1=C(OC)C=CC=C1OC)C2=C(OC)C=CC=C2OC	
C(C1=CC(C)=CC(C)=C1)C2=CC(C)=CC(C)=C2	
C(C1=CC(CC)=CC(CC)=C1)C2=CC(CC)=CC(CC)=C2	
C(C1=CC(C(C)(C)C)=CC(C(C)(C)C)=C1)C2=CC(C(C)(C)C)=CC(C(C)(C)C)=C2	
C(C1=CC(F)=CC(F)=C1)C2=CC(F)=CC(F)=C2	
C(C1=CC(C(F)(F)F)=CC(C(F)(F)F)=C1)C2=CC(C(F)(F)F)=CC(C(F)(F)F)=C2	
C(C1=CC(OC)=CC(OC)=C1)C2=CC(OC)=CC(OC)=C2	
C(C1=CC(C=CC=C2)=C2C=C1)C3=CC(C=CC=C4)=C4C=C3	
C(C1=CC=CC2=C1C=CC=C2)C3=CC=CC4=C3C=CC=C4	
C1=CC=C(C)C=C1	
C1=CC=C(F)C=C1	
C1=CC=C(Cl)C=C1	
C1=CC=C(Br)C=C1	
C1=CC=C(C#N)C=C1	
C1=CC=C(OC)C=C1	
C1=CC=C(O)C=C1	
C1=CC=C([N+](=O)([O-]))C=C1	
C1=CC=C(C(F)(F)(F))C=C1	
C1=CC=C(C(C)(C)(C))C=C1	
CC1=CC=C(C)C=C1	
CC1=CC=C(F)C=C1	
CC1=CC=C(Cl)C=C1	
CC1=CC=C(Br)C=C1	
CC1=CC=C(C#N)C=C1	
CC1=CC=C(OC)C=C1	
CC1=CC=C(O)C=C1	
CC1=CC=C([N+](=O)([O-]))C=C1	
CC1=CC=C(C(F)(F)(F))C=C1	
CC1=CC=C(C(C)(C)(C))C=C1	
C1=CC(C)=CC(C)=C1	
C1=CC(F)=CC(F)=C1	
C1=CC(Cl)=CC(Cl)=C1	
C1=CC([N+]([O-])(=O))=CC([N+]([O-])(=O))=C1	
C1=CC(C(F)(F)(F))=CC(C(F)(F)(F))=C1	
C1=CC(OC)=CC(OC)=C1	
C1=CC(C(C)(C)(C))=CC(C(C)(C)(C))=C1	
C1=C(C)C=CC=C1(C)	
C1=C(C)C=C(C)C=C1(C)	
C1=C(C(C)(C))C=C(C(C)(C))C=C1(C(C)(C))	
C1=C(Cl)C=C(Cl)C=C1(Cl)	
C1=C(C)C=C(C(C)(C)(C))C=C1(C)	
C1=CC(F)=C(F)C(F)=C1	
C1=C(F)C=CC=C1(F)	

C1=C(C)C(C)=C(C)C(C)=C1(C)
C1=C(F)C(F)=C(F)C(F)=C1(F)
CC1=CC(C)=CC(C)=C1
CC1=CC(F)=CC(F)=C1
CC1=CC(Cl)=CC(Cl)=C1
CC1=CC([N+]([O-])(=O))=CC([N+]([O-])(=O))=C1
CC1=CC(C(F)(F)(F))=CC(C(F)(F)(F))=C1
CC1=CC(OC)=CC(OC)=C1
CC1=CC(C(C)(C)(C))=CC(C(C)(C)(C))=C1
CC1=C(C)C=CC=C1(C)
CC1=C(F)C=CC=C1(F)
CC1=C(C)C=C(C)C=C1(C)
CC1=C(C(C)(C))C=C(C(C)(C))C=C1(C(C)(C))
CC1=C(Cl)C=C(Cl)C=C1(Cl)
CC1=C(C)C=C(C(C)(C)(C))C=C1(C)
CC1=CC(F)=C(F)C(F)=C1
CC1=C(C)C(C)=C(C)C(C)=C1(C)
CC1=C(F)C(F)=C(F)C(F)=C1(F)
C1=CC=CC2=C1C=CC=C2
C1=CC=C(C=CC=C2)C2=C1
CC1=CC=CC2=C1C=CC=C2
CC1=CC=C(C=CC=C2)C2=C1
CN(C)CC1=CC=CC=C1
C1=CC2=C(C=CC=C2)C3=CC=CC=C31
C1=C(C=CC=C2)C2=CC3=C1C=CC=C3
CC1=C(C=CC=C2)C2=CC3=C1C=CC=C3
C1=C2C3=C(C=CC4=CC=CC(C=C2)=C43)C=C1
CC1=C2C3=C(C=CC4=CC=CC(C=C2)=C43)C=C1
[Si](C1=CC=CC=C1)(C2=CC=CC=C2)C3=CC=CC=C3
C1=CC(C(C)(C)C)=C(OC)C(C(C)(C)C)=C1
C1=C(C2CCCCC2)C=C(C3CCCCC3)C=C1C4CCCCC4
C1=N[C@@H](C2=CC=CC=C2)[C@H](C3=CC=CC=C3)N1(S(=O)(=O)(CC))
C1=N[C@@H](C2=CC=CC=C2)[C@H](C3=CC=CC=C3)N1(S(=O)(=O)(C(C)(C)))
C1=N[C@@H](C2=CC=CC=C2)[C@H](C3=CC=CC=C3)N1(S(=O)(=O)(C1=CC=C(C)C=C1))
C1=CN(C2=CC=C(C=C3)C4=C2C=CC5=CC=CC3=C45)N=N1
C1=CC=C(C2=CC(C=CC=C3)=C3C=C2)C=C1
C1=CC(C2=C(C)C=C(C)C=C2C)=CC(C3=C(C)C=C(C)C=C3C)=C1
C1=CC(C2=CC=CC=C2)=CC(C3=CC=CC=C3)=C1
C1=C(C(F)(F)F)C=C(C(F)(F)F)C=C1
[Si](C1=CC=C(C(C)(C)C)C=C1)(C2=CC=C(C(C)(C)C)C=C2)C3=CC=C(C(C)(C)C)C=C3
C1=CC=C(C2CCCCC2)C=C1
C1=CC=C(C2=CC(C(F)(F)F)=CC(C(F)(F)F)=C2)C=C1
C1=CC=CC(COC)=C1
C1=CC(C)=C(OC(C)C)C(C)=C1
C1=C(C(C)C)C=C(C2=CC=C(C(C)(C)C)C=C2)C=C1C(C)C
C1=C(Cl)C=C(Cl)C=C1Cl
C1=C(C=CC=C2)C2=C(C3=CC(C=CC=C4)=C4C=C3)C5=C1C=CC=C5
C1=CC(C2=CC(C(F)(F)F)=CC(C(F)(F)F)=C2)=CC(C3=CC(C(F)(F)F)=CC(C(F)(F)F)=C3)=C1
C1=CC=C(S(F)(F)(F)(F)F)C=C1
C1=C(C2=CC(C=CC=C3)=C3C=C2)C=CC=C1
C1=C(OCC)C=CC(C)=C1
C1=C(F)C=C(OC)C=C1F
C1=C(OC)C=CC=C1OC
C1=CC(C2=CC=C(OC)C=C2)=CC(C3=CC=C(OC)C=C3)=C1
C1=C(OC(F)(F)F)C=CC=C1
[Si](C)(C1=CC=CC=C1)C2=CC=CC=C2
C1=CC(C2=CC(C=CC=C3)=C3C=C2)=CC=C1
C1=CC=CC=C1(C(F)(F)F)
C1=C2C3CCC(CC3)C2=CC4=C1C5CCC4CC5
C12C[C@@H](C[C@H]3C2)C[C@@H](C3)C1
C1=C(C(C)C)C=C(C23C[C@@H](C[C@H]4C3)C[C@@H](C4)C2)C=C1C(C)C
C1=CC([N+]([O-])=O)=C(C)C([N+]([O-])=O)=C1
C1=CN(C2=C(F)C=CC=C2)N=N1
C1=CC=C(C2=C(C=CC=C3C4=C5C=CC=C4)C3=C5C=C2)C=C1
C1=CC=CC=C1CCC2=CC=CC=C2
C1=CC=CC([N+]([O-])=O)=C1
C#CC1CCCCC1
C1=C(Cl)C=C(Cl)C=C1
C1=CC=C(C2=CC(C(C)(C)C)=CC(C(C)(C)C)=C2)C=C1
C1=CC=CC=C1[N+]([O-])=O
C1=CC(C2=C(C=CC=C3)C3=C(C=CC=C4)C4=C2)=CC=C1
C1=CC(S(F)(F)(F)(F)F)=CC(S(F)(F)(F)(F)F)=C1
C#CC1=CC=C(C(F)(F)F)C=C1
[Si](C)(C)C(C)(C)C
C#C[Si](CC)(CC)CC
C#CC1=C(C(F)(F)F)C=CC=C1
C#CC1=CC=C([N+]([O-])=O)C=C1
C#CC1=CC=C(OC)C=C1
C#C[Si](C(C)C)(C(C)C)C(C)C
C#CC1=C([N+]([O-])=O)C=CC=C1
C1=C(C(C)C)C=C(C2=C(C=CC=C3)C3=CC4=C2C=CC=C4)C=C1C(C)C

5. Generality Probing Set

Table S6. Combinations of SMILES of SubA and SubB included in the Generality Probing Set (GPS), along with their location in the 2D t-SNE plot of the substrate scope.

#	SubA SMILES	SubB SMILES	Dim1	Dim2
1	[N-]=[N+]=Nc1cccc2[nH]cc(CCN)c12	COC(=O)/C=C/COc1c(Cl)cccc1C=O	0.84	-0.77
2	C=CCNCCc1c[nH]c2ccccc12	O=Cc1cc(Cl)ccc1O	-9.51	1.08
3	CC(C)[C@@H]1CC[C@@H](C)C[C@H]1OC(=O)NCC c1c[nH]c2ccccc12	COC(=O)/C=C/CN(c1ccccc1C=O)S(=O)(=O)c1ccc(C)cc1	-2.60	-6.35
4	NCCc1c[nH]c2cc(O)c(O)c(F)c12	Cc1ccccc1C=O	6.94	5.11
5	CCCCc1ccc2[nH]cc(CCN)c2c1	CCC(=O)C(C)=O	1.27	5.21
6	CCNCCc1c[nH]c2ccc(OC)cc12	COC(=O)/C=C/COc1ccccc1C=O	-4.42	3.11
7	NCCc1c[nH]c2ccc(Cc3ccccc3)cc12	O=Cc1ccc(C(F)(F)F)cc1	1.32	9.58
8	CCOC(=O)CCCc1ccc2[nH]cc(CCN)c2c1	O=Cc1cc2ccccc2s1	8.00	-2.07
9	CCNCCc1c[nH]c2ccc(OC)cc12	O=Cc1c[nH]c2cccc(C(=O)O)c12	3.78	-5.76
10	COc1cc2c(CCN)c[nH]c2cc1O	O=Cc1ccccc1	5.75	8.47
11	Oc1ccc2[nH]cc(CCNCc3ccccc3)c2c1	O=CCOCc1ccccc1	-6.18	-4.45
12	Cc1ccc(C)c2c(CCN)c[nH]c12	CCn1c2ccccc2c2cc(C=O)ccc21	6.54	2.55
13	Cc1cc(OCc2ccccc2)cc2c(CCN)c[nH]c12	CCCC(=O)C(=O)OCC	-1.24	6.87
14	NCCc1c[nH]c2c(F)c(F)c(O)cc12	COc1ccc(C=O)c(F)c1	12.05	2.40
15	COC(=O)c1ccc(OC)c2[nH]cc(CCN)c12	CCC(=O)C(=O)NC1CCCCC1	-2.17	0.60
16	C=CCNCCc1c[nH]c2ccc(F)cc12	C=C[C@H]1[C@H](O[C@@H]2O[C@H](CO)[C@@H](O)[C@H](O)[C@H]2O)OC=C(C(=O)OCCCNC(=O)OC(C)(C)C)[C@H]1CC=O	-6.94	-8.29
17	Cc1cc(C)c(CNCCc2c[nH]c3ccccc23)c(C)c1	O=CC1=NCCS1	-7.28	2.78
18	NCCc1c[nH]c2c(O)cc(O)c(F)c12	O=CC(c1ccccc1)c1ccccc1	10.88	-1.34
19	COc1ccc2[nH]cc(CCNC(C)C)c2c1	C=C=C(CCC=O)c1ccc(Cl)cc1	0.35	-5.74
20	NCCc1c[nH]c2cc(Br)ccc12	O=Cc1cccc2ccccc12	2.87	-8.30
21	COC(=O)c1ccc(OC)c2[nH]cc(CCN)c12	CCOc1ccccc1C=O	5.38	-0.05
22	COC(=O)CCNCCc1c[nH]c2ccccc12	C#CC(=O)OC	-9.14	-3.33
23	COc1ccc2[nH]cc(CCNC(C)C)c2c1	O=Cc1ccccc1C#Cc1ccccc1	-2.60	-3.16
24	Cc1cc(C)c(CNCCc2c[nH]c3ccc(C)cc23)c(C)c1	O=Cc1ccccc1C#Cc1ccccc1	-5.37	-1.75
25	COc1cccc2[nH]cc(CCNC/C=C/COC(=O)OC(C)(C)C)c12	O=Cc1ccc(O[C@H]2O[C@H](COCc3ccccc3)[C@@H](OCc3ccccc3)[C@@H](OCc3ccccc3)[C@H]2OCc2ccccc2)cc1	-0.85	-11.38
26	Cc1cc(OCc2ccccc2)cc2c(CCN)c[nH]c12	COc1ccc2cc(C=O)ccc2c1	9.91	5.76
27	CC(C)(C)c1ccc2[nH]cc(CCN)c2c1	CCC(C)C=O	3.50	3.49
28	NCCc1c[nH]c2c(F)c(F)c(F)c(F)c12	COC(=O)C(=O)CCCO[Si](C)(C)C(C)(C)C	0.48	2.67
29	NCCc1c[nH]c2cc(Cl)c(F)cc12	O=Cc1cccc(C(F)(F)F)c1	2.92	8.03
30	COc1cc2c(CCNOCSC)c[nH]c2cc1Br	O=Cc1cc(-c2ccccc2)on1	2.76	-2.77
31	CCOC(=O)CCNCCc1c[nH]c2ccccc12	C=C=C(CCC=O)C(C)(C)C	-11.45	0.18
32	CCOc1ccc2ccccc2c1S(=O)NCCc1c[nH]c2ccccc12	C=C[C@H]1[C@H](O[C@@H]2O[C@H](COC(C)=O)[C@@H](OC(C)=O)[C@H](OC(C)=O)[C@H]2OC(C)=O)OC =C(C(=O)OC)[C@@H]1CC=O	-4.49	-11.40
33	COc1ccc2[nH]cc(CCN)c2c1	CCOC(=O)CC(=O)C(=O)O	-2.66	8.80
34	[N-]=[N+]=Nc1cccc2[nH]cc(CCN)c12	O=CC1CC1	6.81	-6.11
35	NCCc1c[nH]c2c(F)cc(F)cc12	O=CC(=O)O	8.54	0.45
36	O=[N+]([O-])c1ccc(CNCCc2c[nH]c3ccccc23)cc1	CC(C)(O)[C@@H]1CC[C@](C)(CC=O)O1	-7.79	-0.57
37	COc1ccc2[nH]cc(CCN)c2c1	COC(=O)C1=CO[C@@H](OCC(C)C)C[C@H]1CC=O	-3.06	-9.18
38	NCCc1c[nH]c2ccc(OC(=O)c3ccncc3)cc12	C=CCCCC(=O)C(=O)OC	-1.45	4.01
39	NCCc1c[nH]c2c(O)cc(O)c(O)c12	CC(C)(C)OC(=O)CC[C@@H](C=O)n1cccc1	-0.24	-3.65
40	NCCc1c[nH]c2ccc(Br)cc12	O=Cc1ccccc1O	4.17	6.21
41	Cc1ccc2c(CCN)c[nH]c2c1Br	O=Cc1ccc(=O)[nH]c1	8.68	8.95
42	COc1cc2c(CCNOCSC)c[nH]c2cc1Br	CCSC(CCC=O)(SCC)C(=O)OC	-1.08	0.15
43	COc1ccc2[nH]cc(CCN)c2c1	CC(=O)C(=O)NCc1ccccc1	-4.07	0.32
44	COc1cc2c(CCNOCSC)c[nH]c2cc1Br	C=C=CCN(CC=O)C(=O)OC(C)(C)C	3.00	0.66
45	Clc1ccc2[nH]cc(CCNCc3ccccc3)c2c1	O=Cc1cn(S(=O)(=O)c2ccccc2)c2ncccc12	-0.09	-8.19
46	NCCc1c[nH]c2cc(O)ccc12	O=Cc1ccc2c(c1[N+](=O)[O-])OCO2	0.83	12.02
47	NCCc1c[nH]c2cc(Cl)ccc12	O=Cc1ccoc1	3.99	10.69
48	COc1ccc2[nH]cc(CCN)c2c1	CCC(=O)C(=O)NCC(=O)OC(C)(C)C	-4.72	5.90
49	COc1ccc([N+](=O)[O-])c2c(CCN)c[nH]c12	O=Cc1ccnc2ccccc12	5.21	-3.31
50	NCCc1c[nH]c2c(Br)cccc12	O=Cc1ccc(Br)cc1O	9.88	3.00

6. Evolutionary Experiments

6.1 Specificity-oriented optimization on the HBD catalyst space

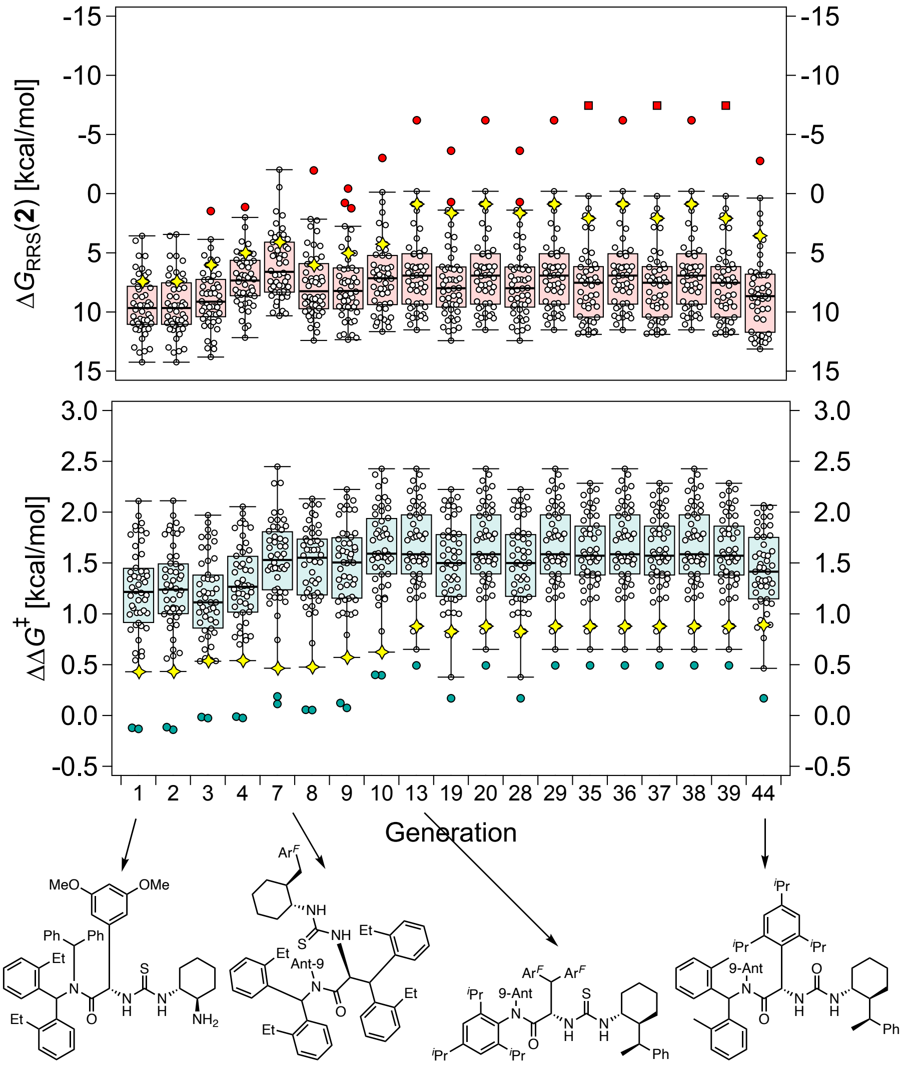

Figure S9. Box-and-whisker chart showing the evolution of $\Delta\Delta G^{\ddagger}$ and $\Delta G_{\mathrm{RRS}}(\mathbf{2})$ of the top individual in the HBD population for selected generations (*i.e.*, when the identity of the best-performing catalyst changes) during the specificity-oriented optimization. Each datapoint corresponds to a reaction in the GPS, the yellow diamond indicates reaction 11 (N_{β}-benzylserotonin + benzyloxyacetaldehyde). Outliers and far outliers are indicated with filled circles and squares, respectively. The optimization targets are $\Delta\Delta G^{\ddagger}$ and $\Delta G_{\mathrm{RRS}}(\mathbf{2})$ of reaction 11.

6.2 Single-objective optimization (SOO) of selectivity on the HBD catalyst space

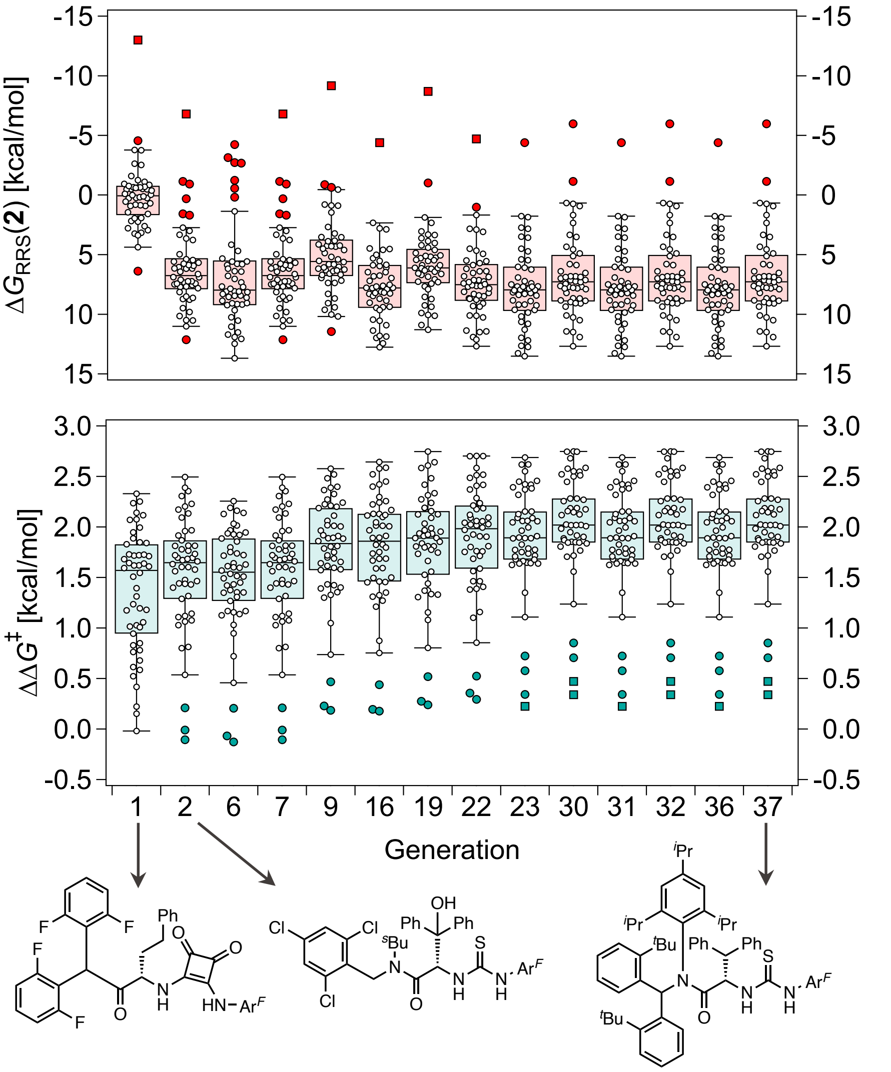

Figure S10. Box-and-whisker chart showing the evolution of $\Delta\Delta G^{\ddagger}$ and $\Delta G_{\mathrm{RRS}}(\mathbf{2})$ of the top individual in the HBD population for selected generations (*i.e.*, when the identity of the best-performing catalyst changes) during the single-objective optimization (SOO) experiment. Each datapoint corresponds to a reaction in the GPS. Outliers and far outliers are indicated with filled circles and squares, respectively.

6.3 Single-objective optimization (SOO) of activity on the HBD catalyst space

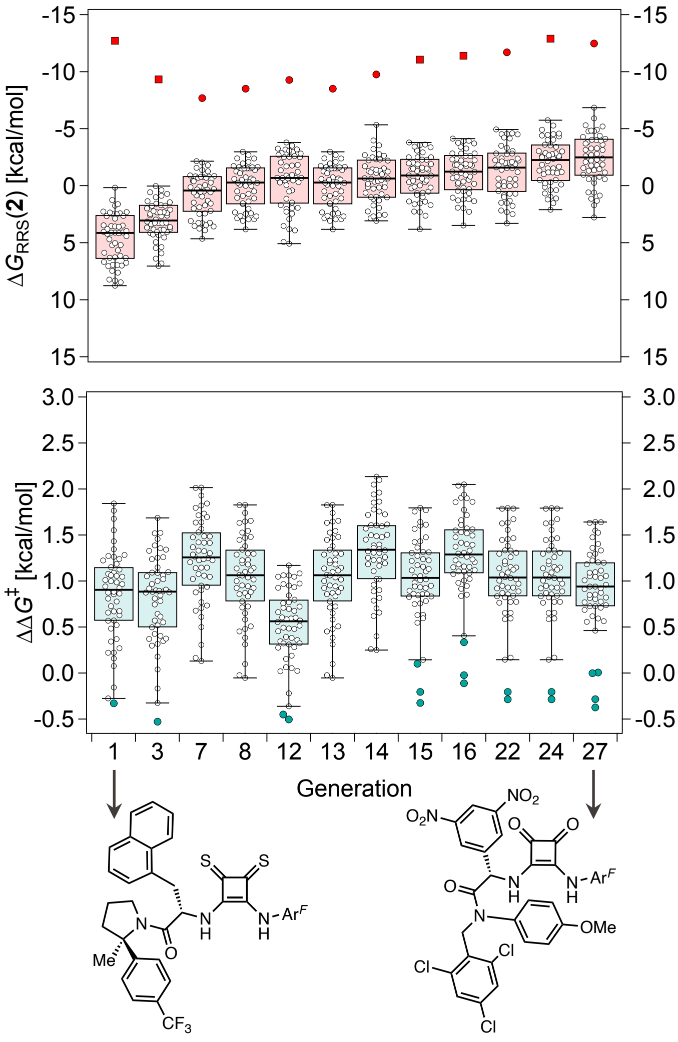

Figure S11. Box-and-whisker chart showing the evolution of $\Delta\Delta G^{\ddagger}$ and $\Delta G_{RRS}(\mathbf{2})$ of the top individual in the HBD population for selected generations (*i.e.*, when the identity of the best-performing catalyst changes) during a single-objective optimization experiment where only $\Delta G_{RRS}(\mathbf{2})_{med}$ is optimized. Each datapoint corresponds to a reaction in the GPS. Outliers and far outliers are indicated with filled circles and squares, respectively.

In the single-objective optimization experiment reported in Figure S11, activity [*i.e.*, $\Delta G_{RRS}(\mathbf{2})_{med}$] improves form 4.1 to -2.5 kcal/mol over the course of 27 generations, approaching the volcano peak of -9.0 kcal/mol (Fig. 4D). Conversely, $\Delta\Delta G^{\ddagger}_{med}$ remains equal to 0.9 kcal/mol and does not exceed 1.4 kcal/mol (generation 14), but the difference between the upper and lower whisker significantly decreases. The catalyst scaffold evolves from the thiosquaramide-pyrrolidino moiety in generation 1 to the squaramide-amide-based template in generation 27.

6.4 Multi-objective generality-oriented optimization with activity prioritized

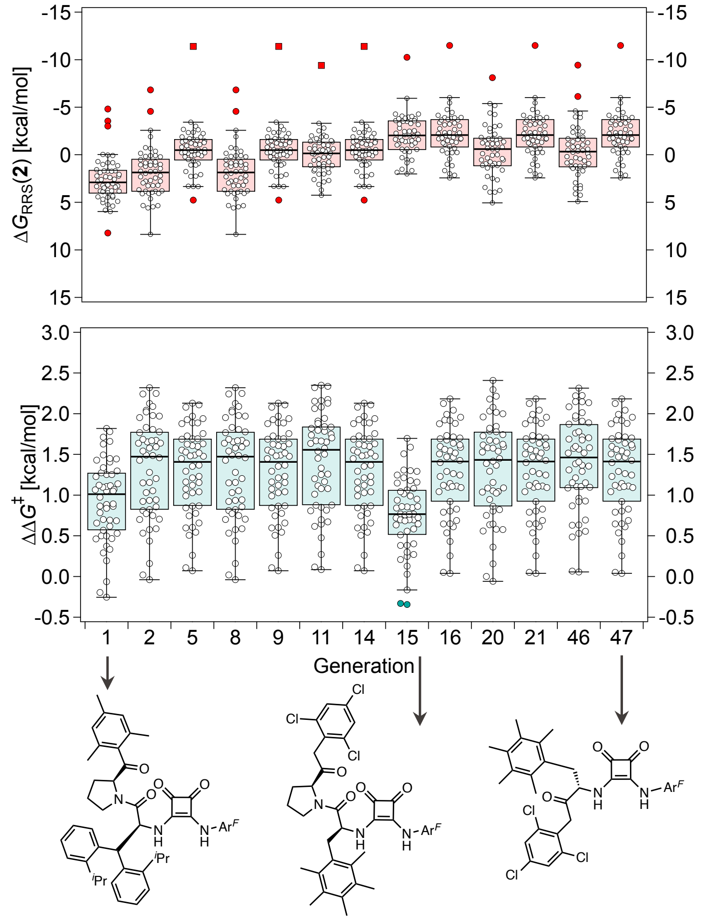

Figure S12. Box-and-whisker chart showing the evolution of $\Delta\Delta G^{\ddagger}$ and $\Delta G_{\mathrm{RRS}}(\mathbf{2})$ of the top individual in the HBD population for selected generations (*i.e.*, when the identity of the best-performing catalyst changes) during a multi-objective optimization experiment with activity prioritized. Each datapoint corresponds to a reaction in the GPS. Outliers and far outliers are indicated with filled circles and squares, respectively.

In this generality-oriented experiment, the targets are scalarized hierarchically using Chimera[15] as follows: first, the median of the activity fitness score f_i of catalyst candidate i across the GPS, defined as $f_i = \exp\left(-\frac{1}{2}\left(\frac{\Delta G_{\mathrm{RRS}}(\mathbf{2}) - x}{5}\right)^2\right)$, a normalized gaussian distribution centered on the target x (-9 kcal/mol, the volcano peak), is maximized. Then, the median selectivity ($\Delta\Delta G^{\ddagger}_{\mathrm{med}}$) across the GPS is maximized with a 10% degradation margin. Finally, the standard deviations of $\Delta\Delta G^{\ddagger}_{\mathrm{med}}$ and median f_i are minimized with a 25% compromise. This experiment differs from the one reported in Fig. 8 by the order of the first two targets, meaning that we allow $\Delta\Delta G^{\ddagger}_{\mathrm{med}}$ to be marginally decreased in order to improve activity.

Over the course of 47 generations, enantioselectivity increases from $\Delta\Delta G^{\ddagger}_{\mathrm{med}}$ = 1.0 kcal/mol to 1.4 kcal/mol, while activity simultaneously improves from $\Delta G_{\mathrm{RRS}}(\mathbf{2})_{\mathrm{med}}$ = 2.9 kcal/mol to -2.1 kcal/mol (*i.e.*, closer to the volcano peak of -9.0 kcal/mol). Compared to the experiment reported in Fig. 8, by inverting the order of priority of the two targets NaviCatGA explores candidates with higher median activity but lower median selectivity: the top organocatalyst found at the end of the evolutionary experiment lacks the amide-based template [–C(=O)NR$_2$] which is associated with high generality, as evinced by the number of reactions in the GPS with $\Delta\Delta G^{\ddagger}$ < 1 kcal/mol.

6.5 Specificity-oriented optimization on GPS reaction 13

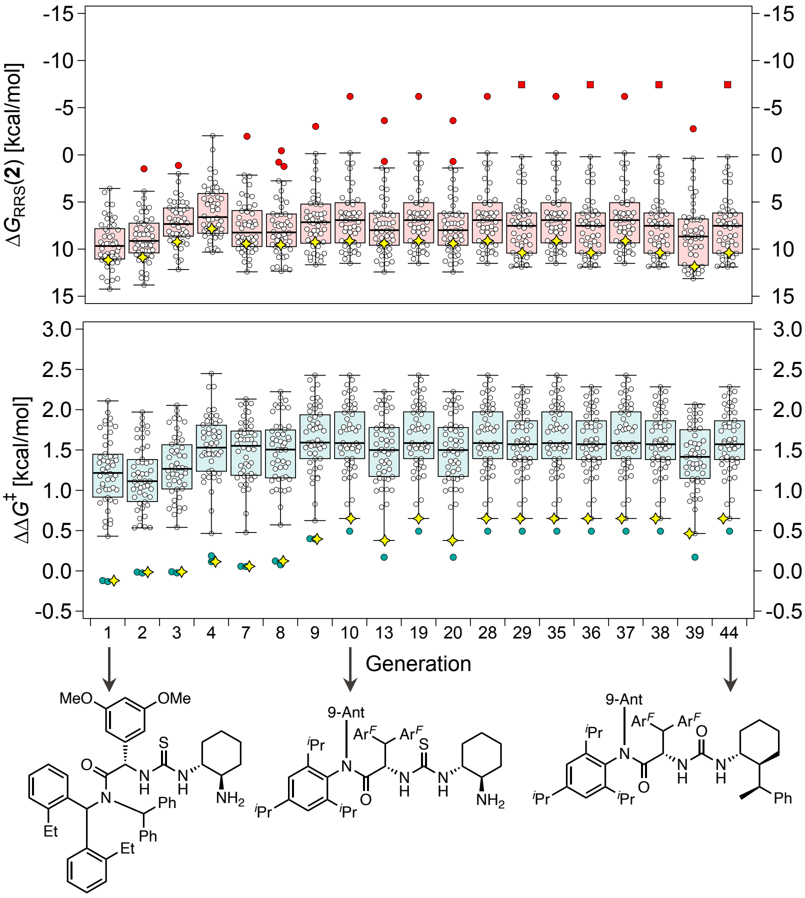

Figure S13. Box-and-whisker chart showing the evolution of $\Delta\Delta G^{\ddagger}$ and $\Delta G_{\mathrm{RRS}}(\mathbf{2})$ of the top individual in the HBD population for selected generations (*i.e.*, when the identity of the best-performing catalyst changes) during a specificity-oriented optimization experiment. Each datapoint corresponds to a reaction in the GPS, the yellow diamond indicates reaction 13 [2-(5-(benzyloxy)-7-methyl-1*H*-indol-3-yl)ethan-1-amine + ethyl 2-oxopentanoate). Outliers and far outliers are indicated with filled circles and squares, respectively. The optimization targets are $\Delta\Delta G^{\ddagger}$ and $\Delta G_{\mathrm{RRS}}(\mathbf{2})$ of reaction 13.

Figure S13 reports another specificity-oriented evolutionary experiment performed on the HBD catalyst space where we wish to simultaneously optimize the activity [$\Delta G_{\mathrm{RRS}}(\mathbf{2})$] and enantioselectivity [$\Delta\Delta G^{\ddagger}$] of reaction 13 in the GPS. This Pictet–Spengler condensation features an unprotected *β*–arylethylamine (SubA) substituted at the 7-position of the indole ring, which has been found to be associated with low stereoselectivity due to the disruption of a key non-covalent interaction between the catalyst's amide O and the indole N–H (see Figures 10 and 11).

Over the course of 44 generations, $\Delta\Delta G^{\ddagger}$ reaches the value of 0.7 kcal/mol, which is slightly higher than that predicted for the top organocatalyst from generation 32 in the multi-objective, generality-oriented experiment (0.4 kcal/mol, Fig. 8 and Fig. 10) or for the one from generation 37 in the single-objective experiment (0.5 kcal/mol, Fig. 9 and Figure S10). Conversely, the catalyst found in this NaviCatGA run is less general ($\Delta\Delta G^{\ddagger}_{\mathrm{med}}$ = 1.6 kcal/mol) than from the Fig. 8 (1.9 kcal/mol) or from the SOO experiment (2.0 kcal/mol). Despite the improvement in predicted enantioselectivity, this transformation remains essentially an outlier in the GPS (the yellow diamond corresponding to reaction 13 lies on the lower whisker of the box plot of generation 44, Figure S13), indicating that HBD catalysts possessing the amide template [–C(=O)NR$_2$] are ill-suited to impart high stereoselectivity in condensations involving 7-indolyl substituents. The best organocatalyst from generation 44 features a cyclohexyl urea motif with an unusual ethylbenzene substituent (also found in the specificity-oriented optimization of

reaction 11, Figure S9) and bulky substituents (9-anthracenyl, 2,4,6-^{i}Pr-C_6H_2) on the amide scaffold. Correspondingly, the activity of this organocatalyst, both in terms of $\Delta G_{\text{RRS}}(\mathbf{2})_{\text{med}}$ (7.5 kcal/mol) and $\Delta G_{\text{RRS}}(\mathbf{2})$ of reaction 13 (10.4 kcal/mol) is low, owing to the poor hydrogen-bonding ability of the urea motif.

7. Data Availability

Data can be found on the Materials Cloud (https://doi.org/10.24435/materialscloud:z7-ev) and is structured as follows:

- Pictet-Spengler_Database_DeltaDeltaG.csv: SMILES strings of Cat, Co-cat, SubA, SubB, Solvent, and corresponding $\Delta\Delta G^{\ddagger}$ value of the 820 Pictet–Spengler reactions in the database;
- Pictet-Spengler_Database_DeltaG_Int2.csv: SMILES strings of Cat, Co-cat, SubA, SubB, and corresponding relative Gibbs free energy of catalytic cycle intermediate 2 (DeltaG_2) of the 703 datapoints in the database of Pictet–Spengler reactions;
- Pictet-Spengler_Database_DeltaG_Int2_Gibbs_Free_Energies.csv: absolute quasi-harmonic Gibbs free energies of Cat, Co-cat, SubA, and SubB used to calculate $\Delta G_{\mathrm{RRS}}(\mathbf{2})$ (703 datapoints);
- Pictet-Spengler_Database_SRS_Gibbs_Free_Energies.csv: absolute and relative quasi-harmonic Gibbs free energies of Cat, Co-cat, SubA, SubB, Product, Int1, Int1B, Int2, Int3, TS1, TS2, and TS3 of the reactions used to construct the TOF-molecular volcano plot (Scaling Relationships Set, SRS, 44 datapoints);
- Pictet-Spengler_Database_Yield.csv: SMILES strings of Cat, Co-cat, SubA, SubB, and Product of the subset of 295 reactions with reported experimental yield;
- Pictet-Spengler_Database_tSNE.csv: SMILES strings of Cat, Co-cat, SubA, SubB, and Product of the 820 Pictet–Spengler reactions in the database, along with the corresponding %*ee* and $\Delta\Delta G^{\ddagger}$ values, the reaction temperature, time, and percentage yield (when reported), and the dimensions of the 2D t-SNE plot;
- Substrate_Scope_Combinatorial_tSNE.csv: SMILES strings of SubA and SubB for the substrate scope, along with the dimensions of the 2D t-SNE plot (97729 datapoints); type_label 1 = organocatalytic, 2 = Reaxys®, 3 = combinatorial;
- Substrate_Scope_GPS_tSNE.csv = SMILES strings of SubA and SubB combinations in the Generality Probing Set, along with the dimensions of the 2D t-SNE plot (50 datapoints).

The XYZ_Structures folder contains the following:

- Pictet-Spengler_Database_Catalysts: DFT-optimized XYZ structures of all catalysts in the Pictet–Spengler database;
- Pictet-Spengler_Database_Co-catalysts: DFT-optimized XYZ structures of HOAc and BzBr co-catalysts;
- Pictet-Spengler_Database_Int2: DFT-optimized XYZ structures of all Int2 catalytic cycle intermediates in the Pictet–Spengler database (703 datapoints);
- Pictet-Spengler_Database_SRS: DFT-optimized XYZ structures of the stationary points along the potential energy surface of the reactions in the SRS;
- Pictet-Spengler_Database_SubA: DFT-optimized XYZ structures of all SubA in the Pictet–Spengler database;
- Pictet-Spengler_Database_SubB: DFT-optimized XYZ structures of all SubB in the Pictet–Spengler database.

8. References

1 M. D. Wodrich, B. Sawatlon, M. Busch and C. Corminboeuf, The Genesis of Molecular Volcano Plots, *Acc. Chem. Res.*, 2021, **54**, 1107–1117.

2 L. van der Maaten and G. Hinton, Visualizing Data using t-SNE, *J. Mach. Learn. Res.*, 2008, **9**, 2579–2605.

3 R. Laplaza, S. Das, M. D. Wodrich and C. Corminboeuf, Constructing and interpreting volcano plots and activity maps to navigate homogeneous catalyst landscapes, *Nat. Protoc.*, 2022, **17**, 2550–2569.

4 S. Kozuch and S. Shaik, How to Conceptualize Catalytic Cycles? The Energetic Span Model, *Acc. Chem. Res.*, 2011, **44**, 101–110.

5 T. Lynch-Colameta, S. Greta and S. A. Snyder, Synthesis of aza-quaternary centers via Pictet–Spengler reactions of ketonitrones, *Chem. Sci.*, 2021, **12**, 6181–6187.

6 J. Seayad, A. M. Seayad and B. List, Catalytic Asymmetric Pictet−Spengler Reaction, *J. Am. Chem. Soc.*, 2006, **128**, 1086–1087.

7 R. Andres, Q. Wang and J. Zhu, Divergent Asymmetric Total Synthesis of (−)-Voacafricines A and B, *Angew. Chem. Int. Ed.*, 2023, **62**, e202301517.

8 A. Mauger, M. Jarret, A. Tap, R. Perrin, R. Guillot, C. Kouklovsky, V. Gandon and G. Vincent, Collective Total Synthesis of Mavacuran Alkaloids through Intermolecular 1,4-Addition of an Organolithium Reagent, *Angew. Chem. Int. Ed.*, 2023, **62**, e202302461.

9 K. Jorner and L. Turcani, MORFEUS, 2022, https://digital-chemistry-laboratory.github.io/morfeus/.

10 J. Werth and M. S. Sigman, Connecting and Analyzing Enantioselective Bifunctional Hydrogen Bond Donor Catalysis Using Data Science Tools, *J. Am. Chem. Soc.*, 2020, **142**, 16382–16391.

11 M. H. Samha, J. L. H. Wahlman, J. A. Read, J. Werth, E. N. Jacobsen and M. S. Sigman, Exploring Structure–Function Relationships of Aryl Pyrrolidine-Based Hydrogen-Bond Donors in Asymmetric Catalysis Using Data-Driven Techniques, *ACS Catal.*, 2022, **12**, 14836–14845.

12 C. B. Santiago, J.-Y. Guo and M. S. Sigman, Predictive and mechanistic multivariate linear regression models for reaction development, *Chem. Sci.*, 2018, **9**, 2398–2412.

13 A. A. Schoepfer, R. Laplaza, M. D. Wodrich and C. Corminboeuf, Reaction-Agnostic Featurization of Bidentate Ligands for Bayesian Ridge Regression of Enantioselectivity, *ChemRxiv*, 2023, DOI:10.26434/chemrxiv-2023-pknnt.

14 R. Pollice and P. Chen, A Universal Quantitative Descriptor of the Dispersion Interaction Potential, *Angew. Chem. Int. Ed.*, 2019, **58**, 9758–9769.

15 F. Häse, L. M. Roch and A. Aspuru-Guzik, Chimera: enabling hierarchy based multi-objective optimization for self-driving laboratories, *Chem. Sci.*, 2018, **9**, 7642–7655.
